# Evaluation of Blood Biomarkers Associated with Risk of Malnutrition in Older Adults: A Systematic Review and Meta-Analysis

**DOI:** 10.3390/nu9080829

**Published:** 2017-08-03

**Authors:** Zhiying Zhang, Suzette L. Pereira, Menghua Luo, Eric M. Matheson

**Affiliations:** 1 Department of Kinesiology and Community Health, University of Illinois at Urbana-Champaign, Champaign, IL 61820, USA; zzhang26@illinois.edu or zhiying.zhang@abbott.com; 2 Abbott Nutrition Research and Development Asia-Pacific Center, 138668 Singapore, Singapore; 3 Abbott Nutrition Research and Development, Columbus, OH 43219, USA; Menghua.Luo@abbott.com; 4 Department of Family Medicine, Medical University of South Carolina, Charleston, SC 29412, USA; matheson@musc.edu

**Keywords:** malnutrition, biomarker, nutrition screening tools, BMI, albumin, prealbumin, hospitalized patients

## Abstract

Malnutrition is a common yet under-recognized problem in hospitalized patients. The aim of this paper was to systematically review and evaluate malnutrition biomarkers among order adults. Eligible studies were identified through Cochrane, PubMed and the ProQuest Dialog. A meta-regression was performed on concentrations of biomarkers according to malnutrition risks classified by validated nutrition assessment tools. A total of 111 studies were included, representing 52,911 participants (55% female, 72 ± 17 years old) from various clinical settings (hospital, community, care homes). The estimated BMI (*p* < 0.001) and concentrations of albumin (*p* < 0.001), hemoglobin (*p* < 0.001), total cholesterol (*p* < 0.001), prealbumin (*p* < 0.001) and total protein (*p* < 0.05) among subjects at high malnutrition risk by MNA were significantly lower than those without a risk. Similar results were observed for malnutrition identified by SGA and NRS-2002. A sensitivity analysis by including patients with acute illness showed that albumin and prealbumin concentrations were dramatically reduced, indicating that they must be carefully interpreted in acute care settings. This review showed that BMI, hemoglobin, and total cholesterol are useful biomarkers of malnutrition in older adults. The reference ranges and cut-offs may need to be updated to avoid underdiagnosis of malnutrition.

## 1. Introduction

Malnutrition is highly prevalent in hospitalized patients yet remained underdiagnosed, especially in the frail, elderly population. Only 3 to 5% of hospitalized population is diagnosed with malnutrition, although it is estimated that 30–60% of the hospitalized population are malnourished [1,2,3,4]. Disease-associated malnutrition, if under-recognized, could cause significant economic burdens [5]. In the absence of a universally accepted definition of malnutrition and a “gold-standard” for its diagnosis, many nutrition screening and assessment tools have been developed. A number of them have been validated and recommended, including Nutritional Risk Screening 2002 (NRS-2002), Mini Nutritional Assessment (MNA), Malnutrition Universal Screening Tool (MUST), and Subjective Global Assessment (SGA) [6,7,8]. However, these tools use different criteria and cut-offs and were designed for different purposes and populations, thus are not uniformly applied across clinical situations. Furthermore, nutrition assessment based on these tools could be a complex process because they often involve assessment of nutritional intake, changes in body composition, signs or symptoms of nutritional deficiency or excess. Sometimes, the assessments can be subjective per personal experience; reproducing the data becomes a challenge.

Because of this complexity, practitioners have sought a rapid, more convenient laboratory means, usually involving serum biochemicals, measured as part of routine blood tests, to identify patients at risk of malnutrition. In clinical practice, blood diagnostics also have the advantage to ensure immediate nutrition assessment and prompt intervention for patients who are malnourished or at risk of malnutrition. Protein markers, such as albumin and prealbumin (i.e., transthyretin), were once the standard blood biomarkers for diagnosing malnutrition during hospital admissions. However, studies revealed that these are negative acute phase protein whose serum levels are affected not only by nutrition status, but also by many other factors such as inflammation, infection, liver damage, fluid status, etc. [9,10,11]. Therefore, these blood biomarkers are no longer recommended for identifying malnutrition by the AND (Academy of Nutrition and Dietetics) and A.S.P.E.N. (American Society for Parenteral and Enteral Nutrition) [4]. Instead, A.S.P.E.N. and AND proposed that malnutrition is to be diagnosed in clinical practice if an adult meets at least 2 of the following 6 criteria: (1) insufficient energy intake; (2) weight loss; (3) loss of subcutaneous fat; (4) loss of muscle mass; (5) localized or generalized fluid accumulation that may sometimes mask weight loss; and (6) diminished functional status [4]. Nevertheless, it was pointed out that laboratory biochemical tests of the blood could still aid in the determination of the newly proposed etiology-based definition of malnutrition. They can be used to support the presence of systemic inflammatory response and further contribute to the identification of the etiologic basis for the diagnosis of malnutrition.

The correct interpretation of hematology test results and making accurate clinical decisions require well-defined reference values for the respective marker. However, there is still a lack of consensus on the optimal cutoffs and reference ranges of marker levels for evaluation of malnutrition risk among older people. Blood biomarker concentrations often vary with age, sex, race, diet, metabolism, and disease status [12]. The applicability of traditional cut-off values previously set for the general population is questionable for determining malnutrition in older people [13]. Reliance on the conventional threshold values may result in misdiagnosis of malnutrition. Therefore, there is a need to update the reference values of individual biomarkers for older people at different degree of malnutrition risk, based on a large number of subjects from various clinical settings.

Given that blood biomarkers for malnutrition remain widely used in clinical practice, we conducted a comprehensive systematic review and meta-analyses of blood biomarkers commonly used to assess nutrition status in older adults from various clinical settings, including hospitalized inpatients, outpatients, institutionalized elderly and those received home care. The purpose of this study is to summarize publications on blood biomarkers for malnutrition among order adults and to evaluate them against validated nutrition screening tools. We explored the reference ranges of potential biomarkers for subjects at different degree of malnutrition risk, while taking into account the effect of age, gender and disease acuteness. Although it is well-known that malnutrition and sarcopenia (loss of muscle mass along with strength/function loss) may overlap, this review did not attempt to address biomarkers that associated with sarcopenia, mostly due to limitation on the number of malnutrition-related publications that collect body composition data along with strength/functionality data. This review will give an overview of the ability of potential blood biomarkers to identify malnutrition risk among patients with or without stress of acute disease. It provides a general guideline for using blood biomarkers to assess malnutrition that is relevant for subjects in various clinical settings.

## 2. Materials and Methods

The review was planned and conducted following the UK National Health Service Centre for Reviews and Dissemination (CRD) guidance [14], and reported according to the PRISMA guideline (Supplementary Table S8) [15].

### 2.1. Data Sources and Search Strategy

A range of electronic databases were searched using the ProQuest Dialog (PQD) service, including MEDLINE (1950 to present), EMBASE (1974 to present), Embase, Foodline: SCIENCE, Food Science & Technology Abstracts (FSTA), and ProQuest Dissertations and Theses Professional. We also searched PubMed, Cochrane Database of Systematic Reviews and Cochrane Central Register of Controlled Trials. The search strategy for PQD is detailed in Supplementary Table S1. The last search was run on 25 April 2017. No restriction was placed on studies with regard to year of publication and language (providing an English abstract was available). The bibliographic references of all selected studies and review articles were manually screened for additional eligible studies.

### 2.2. Inclusion Criteria

We included studies evaluating blood levels of biomarkers related to malnutrition in adults from various clinical settings, whether they live in the community or they are in hospital or institutionalized in a long-term care facility. Included studies were required to examine association of blood biomarkers to any of the following validated nutrition screening and assessment tools: Malnutrition Screening Tool (MST), MUST, NRS-2002, Geriatric Nutritional Risk Index (GNRI), Nutritional Risk Index (NRI), Instant Nutritional Assessment (INA), Nutrition Screening Initiative (NSI) and Short Nutritional Assessment Questionnaire (SNAQ), MNA, Mini Nutritional Assessment-Short Form (MNA-SF), SGA, and Detailed Nutritional Assessment (DNA). Studies specifically on patients with kidney disease or cancer were not considered. For studies with multiple publications on the same study population, we selected the study with the largest number of subjects or the most recent publication as the primary report.

### 2.3. Data Extraction

Titles and abstracts resulting from the search strategy were evaluated by two independent investigators. Disagreements were resolved by consensus and, when necessary, by consultation with another investigator. One reviewer performed the data extraction with a second investigator checking the data extraction forms for accuracy and completeness. The studies were grouped according to individual blood biomarkers and the validated nutrition screening tools used. Data were extracted and collated on the following study characteristics: reference, study design, clinical settings (inpatient, outpatient, institutionalized, and home care), subjects (number of subjects, age, and gender ratio), health concerns, nutrition assessment tools, biomarkers, key results, and conclusions. Patients admitted to the intensive care unit (ICU), acute/critical/emergency units, experienced infections, trauma, burns, or post-surgery were considered acute. Disease acuteness was coded as ICU, acute (Y), non-acute (N) or both (B). The primary outcome required was mean and standard deviation (SD) of biomarker values in different malnutrition groups classified by a nutrition assessment tool. Where appropriate, we contacted lead authors for missing data and to clarify suspicious values of biomarker measurements. Outliers of biomarker values were flagged and checked during subsequent statistical analysis.

### 2.4. Quality Assessment

The quality of included studies was assessed using the National Institutes of Health (NIH) Quality Assessment tool for Observational Cohort and Cross-Sectional Studies [16]. The tool was designed to assess the internal validity and risk of bias for observational studies, which contains 14 criteria evaluating the aims of the study, sources of bias, sampling, participation rate, study power, data collection methods, and confounding. The criteria were rated as either yes, no, or “other” (i.e., cannot determine, not reported, or not applicable). An overall rating for the study as “good,” “fair,” or “poor” was provided.

### 2.5. Meta-Analysis

Mixed-effects, multiple linear regression models for meta-analysis were used to compare between-group differences in mean biomarker levels according to malnutrition status defined by validated nutrition screening tools, while adjusting for the effect of age and gender proportion. The heterogeneity among studies was estimated by Q test and *I*^2^ statistic. An *I*^2^ > 50% represented substantial heterogeneity. A fixed-effects model with weighting of the studies was used when there was a lack of significant heterogeneity (*p* > 0.10), while a random-effects model with weighting of the studies was used when there was heterogeneity between studies (*p* ≤ 0.10). We assessed publication bias by visual inspection of the funnel plot and Egger’s test for funnel plot asymmetry. A sensitivity analysis was conducted to determine whether including patients with acute disease would substantially affect the biomarker levels. Two-sided P value was used with an α level of 0.05. all Statistical analyses were performed with R version 3.2.3 (R Foundation for Statistical Computing, Vienna, Austria) with the package metaphor, version 1.9-8 (Wolfgang Viechtbauer, The metafor Package: A Meta-Analysis Package for R).

## 3. Results

### 3.1. Study Characteristics

**Figure 1:**
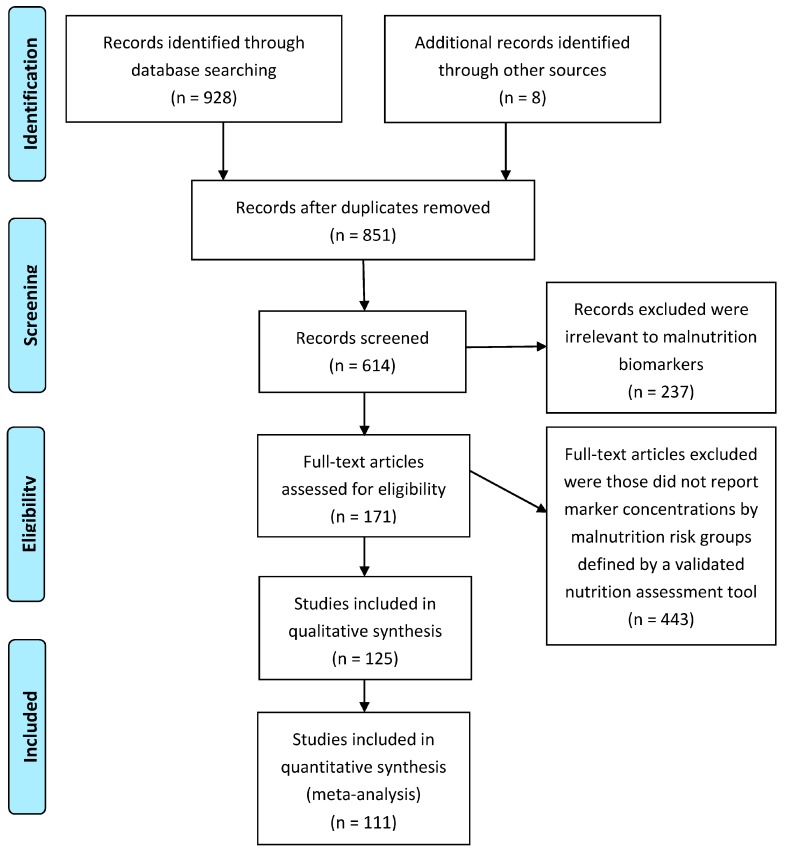
Flow diagram of the search and review process.

After screening 936 publications, 111 studies representing a pool of 52,911 participants (28,988/23,923; female/male) finally met the inclusion criteria. The selection process is illustrated in Figure 1. The mean age of the included subjects was 72 years old with a standard deviation of 17 years. The subjects included hospitalized inpatients (*n* = 39,027), outpatients (*n* = 4159), and those received health care provided at home (*n* = 3400) or in the community (*n* = 6325), of which 4071 are elderly institutionalized in various types of long-term care facilities including nursing home, retirement home, and group homes. As for nutrition assessment tools, MNA was the most frequently used tool for malnutrition assessment, followed by MNA-SF, SGA, GNRI, and NRI. Other tools, such as NRS-2002 and MUST [17,18,19], were used infrequently. SNAQ [20], INA [21], and NSI [22] were used rarely.

### 3.2. Biomarker Characteristics

A total of 43 blood biomarkers were identified in the literature reviewed but only 17 of them had 3 or more studies for meta-analysis. BMI was included as a reference for the analysis because most of the nutrition assessment tools include BMI as a criterion to define malnutrition. The most commonly studied blood biomarker was albumin, followed by hemoglobin (Hb), total cholesterol (Tch), total lymphocyte counts (TLC), prealbumin (PAB), C-reactive protein (CRP), total protein (TP), transferrin (TF), creatinine (Cre), triglycerides (TG), white blood cells (WBC), blood urea nitrogen (BUN), % hematocrit (HT), iron, and estimated glomerular filtration rate (eGFR). The remaining blood biomarkers were evaluated in no more than 3 studies for any of the nutrition assessment tools. In addition to blood biomarkers, BMI is still commonly used as part of the nutrition assessment and has been unanimously reported in almost all the studies. Therefore, it would be helpful to also include BMI as a reference biomarker for the meta-analysis.

### 3.3. Quality Assessment

The overall quality of the included studies was rated as fair (Supplementary Table S2). All studies clearly stated the research objectives and defined populations being studied. Sample sizes were clearly reported in all studies but no justification was provided. Malnutrition risk groups were all identified by a validated nutrition assessment tool, usually categorized into low, medium and high levels, which allows for examination of a dose-dependent relationship. BMI and blood biochemical concentrations were all measured using well-accepted methods, although variations may exist across clinical settings. A majority of the studies represented a cross-sectional design since assessment of nutrition status and analysis of biomarkers were generally carried out within 48 hours of admission for inpatients or at baseline for non-hospitalized subjects. Several studies considered gender or age as confounding variables and presented biomarker levels separately for men and women [22,23,24,25] or for age greater than 65 or not [26]. Correlation coefficients for men and women respectively were reported in 3 studies [23,27,28]. A number of studies also presented regression associations [26,29,30,31,32,33,34] or correlation coefficients [35,36,37,38,39] adjusted for potential confounding variables, including age, gender, BMI, and morbidities. However, no study has presented confounder-adjusted biomarker level estimates. Therefore, the overall quality of the studies was quite consistent with a moderate rating.

### 3.4. Meta-Regression Analysis for Biomarker

There was an evidence of substantial heterogeneity (*p* < 0.001, *I*^2^ > 50%) across studies for all the biomarkers. No publication bias was observed by evaluation of the funnel plots (Supplementary Figure S6). Table 1, Table 2, Table 3, Table 4 and Table 5 and Supplementary Tables S3–S7 present the predicted mean biomarker levels for a population aged 72 years old with half women and half men based on the multivariate meta-regression analyses. Results for subjects of other age groups and gender ratio can also be estimated. Forests plots of BMI, albumin, prealbumin, hemoglobin, and total cholesterol by malnutrition risk groups classified by MNA are shown in Supplementary Figures S1–S5. As expected, BMI performed the best in identifying malnutrition because it screened out not only subjects at high risk of malnutrition as determined by MNA (*p* < 0.001), SGA (*p* < 0.001), and NRS-2002 (*p* < 0.01), but also those at low risk of malnutrition as determined by MNA (*p* < 0.01) and SGA (*p* < 0.01) (Table 1, Table 3, Table 4 and Table 5). The estimated BMIs for each malnutrition risk groups varied among different assessment tools, reflecting the fact that different cut-points of BMI are used for different tools. For subjects at low risk of malnutrition as classified by SGA, a tool that does not include BMI as a criterion, the estimated BMI was 22.92 kg/m^2^, which was lower than that by MNA (25.20 kg/m^2^), NRS-2002 (25.77 kg/m^2^) and GNRI (23.96 kg/m^2^) (Table 1, Table 3, Table 4 and Table 5).

Among all the 17 blood biomarkers, the estimated concentrations of albumin (*p* < 0.001), hemoglobin (*p* < 0.001), total cholesterol (*p* < 0.001), prealbumin (*p* < 0.001) and total protein (*p* < 0.05) for subjects identified by MNA as at high malnutrition risk were statistically lower than those without a malnutrition risk (Table 1). Similar results were observed for that classified by SGA, except for prealbumin, which was not statically significant (Table 3). Compared to subjects of low malnutrition risk identified by NRS-2002, those at high malnutrition risk also had significantly higher hemoglobin (*p* < 0.01) level (Table 4). Since GNRI is based on measurements of serum albumin and weight loss, it was expected that albumin (*p* < 0.001) and prealbumin (*p* < 0.05) were able to identify patients at risk of both high and low malnutrition as assessed by GNRI (Table 5).

The estimated biomarker mean concentrations for each malnutrition risk groups also varied among different assessment tools, with the highest estimates observed for NRS-2002, followed by MNA and SGA. For example, the estimated mean albumin concentration for patients at high risk of malnutrition was 3.42 (95% CI: 3.19, 3.64), 3.31 (95% CI: 3.13, 3.49), and 3.08 (95% CI: 2.84, 3.31) g/dL by NRS-2002, MNA and SGA respectively (Table 1, Table 3 and Table 4). This indicates that using albumin with a cutoff of 3.5 g/dL would fail to identify a proportion of the patients diagnosed to be at high risk of malnutrition using NRS-2002 (Table 4), not to mention those at low malnutrition risk. CRP and TLC failed to distinguish patients at risk of malnutrition defined by any of the tools, although their concentration reflected the presence of inflammatory response, even when acute patients are removed.

A sensitivity analysis indicates that some biomarkers, especially CRP and TLC, were dramatically affected by stress of acute illness (Table 2, Table 3, Table 4 and Table 5). When studies on patients with acute disease were also included, the estimated concentrations of albumin and prealbumin were decreased by more than 5% for groups with malnutrition risk as assessed by NRS-2002 and SGA respectively, although those by MNA were relatively stable (Table 2, Table 3 and Table 4). The reduction on albumin and prealbumin levels were more significant when GNRI was used as the assessment tool for malnutrition, with a more than 10% of decline observed by including patients with acute disease (Table 5). BMI, hemoglobin, total cholesterol, and total protein were not sensitive to acute disease status (Table 2, Table 3, Table 4 and Table 5).

## 4. Discussion

Blood biomarkers, especially albumin, are often used for diagnosis of malnutrition in clinical practice. Yet there is a lack of evidence-based clinical guidance to support their application under specific conditions and settings [96]. The present paper attempted to address this gap. To our knowledge, this is the first systematic review that has evaluated a large number of malnutrition-related blood biomarkers in order adults in order to determine their association with risk of malnutrition as defined by a validated nutrition assessment tool.

Results from our meta-analysis showed that several blood biomarkers, including albumin, prealbumin, hemoglobin, total cholesterol, and total protein, are useful biochemical indicators of malnutrition, even with the presence of chronic inflammation. Inflammation, due to disease or ageing, is an important etiologic factor to the development of malnutrition [97]. Since a majority of the subjects in this study is hospitalized geriatric patients, inflammation is expected to be common among our study population. This is confirmed by the elevated CRP level (>10 mg/L) above normal range in our study, even after patients with acute disease were removed. The inflammation, however, may not be caused by acute virus infection because the concentration of TLC (1.0–3.0 × 10^3^/µL) and WBC (>8 × 10^3^/µL) are within the normal range. While CRP, TLC, and WBC may serve as indicators of inflammatory status, they are not good markers of malnutrition status. As for other blood biomarkers, including transferrin, creatinine, total triglycerides, iron, and % hematocrit, we do not find sufficient evidence to support their use as a marker of malnutrition.

Although BMI as a biomarker is not the focus of our meta-analysis, it is worth presenting the results here given its widespread utilization in clinical practice, Low BMI is commonly accepted to be a criterion for malnutrition diagnosis [98]. It is not surprising that BMI performed better than blood measurements to detect malnutrition defined by validated nutrition screening tools. This is because tools such as the MNA, MUST, and NRS-2002 incorporate BMI as an aid in screening for malnutrition, while SGA includes anthropometric measures that are known to highly correlate with BMI. However, it remains a challenge to find a valid and clinically relevant cut-off value for BMI. Our results support the use of higher BMI cut-off points to identify malnutrition in older people. To warrant identification of all individuals that are at risk of malnutrition, a cut-off of 23 kg/m^2^ for subjects 72 years of age is suggested, which is consistent to a recent ESPEN consensus that suggested using <22 kg/m^2^ in subjects older than 70 years [98]. A simple adoption of the well-accepted BMI cut-off of 18.5 kg/m^2^ provided by World Health Organization (WHO) to define malnutrition would likely fail to identify some patients who are at risk of malnutrition.

Albumin is the most extensively studied proteins for diagnosing malnutrition. Our results support the role of serum albumin as a useful indicator of overall nutrition status for older adults in non-acutely ill states [99]. However, evidence suggests that using a cutoff point of 3.5 g/dL for serum albumin as an indicator of malnutrition may not be suitable for older people, especially those hospitalized elderly. Hypoalbuminemia, often defined as serum albumin concentration <3.5 g/dL, is traditionally regarded as a standard indication of malnutrition. According to the estimated albumin concentrations for subjects without acute disease in our meta-analysis, use of a serum albumin cutoff of 3.5 g/dL as a marker of malnutrition would lead to under-diagnosis of malnutrition defined by all the validated nutrition screening tools (MNA, NRS-2002, MNA-SF, GNRI) except for SGA. Adopting this traditional definition of hypoalbuminemia as indication of malnutrition may only screen out the most severely malnourished ones but not those with lower malnutrition risk.

Similar to albumin, serum prealbumin has also been used as a blood marker of malnutrition. The normal value of prealbumin is 20–40 mg/dL and a mild degree of malnutrition is indicated by a range of 10–20 mg/dL, with serious malnutrition signaled by values below 10 mg/dL [100]. While results from our study suggest that measuring albumin levels is a useful tool to identify malnutrition in non-acute clinical settings, these classification cut-offs may not be appropriate. In the present study, we have observed that only a proportion of the subjects in the high risk group would have a prealbumin concentration below 20 mg/dL, thus leads to underdiagnosis of malnutrition.

The recent etiology-based approach for diagnosis of adult malnutrition recognizes the importance of inflammation in the pathophysiology of malnutrition [4]. In acute healthcare settings, the concentrations of many biomarkers can potentially be altered by many non-nutritional factors, such as infection and inflammation [101]. Our sensitivity analysis confirmed that concentrations of serum albumin and prealbumin were dramatically reduced in response to acute inflammatory stress, acting as “negative acute-phase proteins”. Therefore, measures on these two serum proteins must be interpreted with caution in patients with infection, acute inflammation, or recent trauma. A sensitivity analysis restricted to patients with acute disease showed that albumin concentration for patients with high risk of malnutrition determined by MNA and SGA was not significantly lower than that for those without a risk of malnutrition (data not shown). This observation is consistent with a recent study on post-acute care geriatric patients, whose serum albumin and total protein levels were found to be low regardless of malnutrition or sarcopenia diagnosis [102]. Due to limited number of studies on patients admitted to the acute care wards in our review, further studies are needed to evaluate the validity of using serum albumin or prealbumin in the acute care setting.

Blood hemoglobin, total cholesterol and total protein were also found to be useful markers of adult malnutrition. More importantly, it was observed that these biochemical measures were insensitive to acute disease stress. The normal hemoglobin range is generally defined as 13.5 to 17.5 g/dL of blood for men and 12.0 to 15.5 g/dL for women [103]. The estimated hemoglobin level for our study population is relatively low, even among those characterized as without malnutrition risk (<14.2 g/dL). Our results support the use of a cut-off of <13 g/dL as marker of malnutrition, which is in agreement with the WHO’s lower limit of normal hemoglobin in adults (13 g/dL in men and 12 g/dL in women). Serum cholesterol levels lower than 160 mg/dL, defined as “hypocholesterolemia”, have been considered a reflection of malnutrition [11]. However, using this commonly applied low limit would result in an under-diagnosis of malnutrition, with a large number of at risk patients missed identification. The normal range for total protein in blood fluid is typically between 6.0 and 8.3 g/dL. It should be noted that use of a serum total protein level <6 g/dL as a marker of malnutrition would miss a proportion of subjects determined to be at risk of malnutrition by MNA and SGA.

Combining several biomarkers that are reflective of nutrition status may have the potential to increases sensitivity and specificity. Two studies attempted to combine validated screening tool(s) and/or a blood biomarker to screen malnutrition risk [21,104]. Several studies directly combined one or more blood biomarkers and anthropometric measure(s) to identify malnutrition [105,106,107,108,109,110]. Nutrition indices that combine anthropometric measurements with biochemical indices, such as the prognostic inflammatory and nutritional index [111,112] and the geriatric nutritional risk index (GNRI), have also been proposed for malnutrition assessment. However, none of these has yet been validated as reliable for different clinical settings. We found that GNRI as a marker for malnutrition is sensitive to acute disease stress, due to its reliance on measures of albumin concentration. Future validation studies are required before these nutrition indices can be recommended for use in patients in acute care settings.

Although our findings are based on observational studies, there are several advantages. First, this is the first comprehensive meta-analysis conducted to assess the association between malnutrition risk and blood biomarkers in older adults. Our review covered a wide array of serological markers and provided their reference values by taking into account the potential confounding effect of age, gender, and acute disease stress. Second, a dose-response relationship was observed between biomarker levels and degree of malnutrition risks. Third, we used validated nutrition screening/assessment tools as the standard against which to evaluate the potential of individual blood biomarkers to detect malnutrition risk. We were able to compare and cross-validate results using different tools, including MNA, SGA, NRS-2002, GNRI and MNA-SF. Given that blood biomarkers are widely used in clinical practice, our findings provide useful evidence-based clinical guidance to support their application under specific conditions and settings.

However, there are some limitations that warrant consideration. First, the sample size for each individual marker is unequal. Albumin is the most commonly used blood biomarker for assessing malnutrition, followed by hemoglobin, total cholesterol, total lymphocyte counts, prealbumin, and total protein. Fewer studies evaluated the other blood biomarkers, including transferrin, creatinine, total triglycerides, iron, and % hematocrit, so that the statistical power may be limited. Further investigation with greater numbers would be required to establish whether these blood biomarkers are valid indicators for risk of malnutrition.

Second, our result is based on blood biomarker concentrations measured in only one point of time, either at admission to the hospital, visited a health care provider, or during the pre-surgery examination. Therefore, the diagnostic performance of the individual biomarker is considered an indicator of the general nutrition status at the time when blood sample was taken. A large prospective study with well-controlled time points for blood collection is needed to truly validate these findings. In addition, analysis of the biomarkers on a single diagnostic platform may help determine if the observed variability in the cut points for the biomarkers are truly clinical variability or variations due to differences in the diagnostic assays used across the studies. It would also be useful to determine if the identified markers can change in response to nutrition repletion, which can then be useful markers to determine early benefits of an intervention for malnutrition.

Third, while our study encompassed a large number of subjects from various clinical setting (the community, care homes, hospital), studies specifically on patients with kidney disease or cancer are not included. Such a selection better reflects the general hospitalized/institutionalized population. It also recognizes the fact that many blood biomarkers are affected by kidney malfunction and cancer pathology.

Finally, we would like to point out that although our results provide a useful guideline for using blood biomarkers to assess malnutrition in clinical practice, we do not encourage the sole use of a single biomarker to diagnose malnutrition in a patient. Blood biomarkers should only be used as a complement to a thorough physical examination. A medical diagnosis can only be made through careful consideration of findings from all available information including but not limited to medical and dietary records, physical examination, and laboratory tests.

## 5. Conclusions

In conclusion, this systematic review confirmed that BMI and several blood biochemicals, including albumin, prealbumin, hemoglobin, total cholesterol, and total protein, are useful biomarkers for adult malnutrition, even with the presence of chronic inflammation. However, the reference ranges and cut-offs may need to be updated to avoid under-diagnosis of malnutrition. It confirms that in acute health care settings, albumin and prealbumin must be carefully interpreted because they may be affected by changes brought about by acute disease and the associated inflammation. Future studies should address the important questions of whether these blood biomarker response to nutrition treatment and which biomarker works better. Additionally, efforts can be devoted to develop an algorithm using factors that together are most predictive of risk of malnutrition. In older people, malnutrition and sarcopenia often coexists. Further research is also needed to determine the association of malnutrition biomarkers with sarcopenia and that of other anthropometric measurements such as body muscle mass, mid-arm circumference, calf circumference etc. with the nutrition assessment tools.

## Figures and Tables

**Table 1:** Mean biomarker levels in 3 subgroups of malnutrition risk status defined by MNA, excluded patients with acute disease ^1^.

Marker (Units)	References	*n*	MNA > 23.5 (No Risk) Mean (95% CI)	MNA 17–23.5 (Low Risk) Mean (95% CI)	MNA < 17 (High Risk) Mean (95% CI)	Stats	*I*^2^
BMI (kg/m^2^)	[24,30,32,33,34,35,38,39,40,41,42,43,44,45,46,47,48,49,50,51,52,53,54,55]	4598	27.19 (26.26, 28.11)	25.20 (24.24, 26.17)	20.89 (19.81, 21.96)	LR: *p* < 0.01 HR: *p* < 0.001	93.66%
Albumin (g/dL)	[23,24,28,30,32,33,34,35,38,39,40,41,43,45,46,47,48,49,50,51,52,55,56,57,58,59,60]	6538	3.83 (3.67, 3.99)	3.65 (3.48, 3.81)	3.31 (3.13, 3.49)	LR: *p* < 0.1 HR: *p* < 0.001	98.22%
Hemoglobin (g/dL)	[24,27,28,30,32,33,39,40,41,42,43,44,46,48,50,54,57,58,60]	4526	12.99 (12.60, 13.39)	12.50 (12.11, 12.89)	11.79 (11.33, 12.24)	LR: *p* < 0.1 HR: *p* < 0.001	93.82%
Total Cholesterol (mg/dL)	[23,28,30,34,38,39,41,43,46,47,48,50,52,54,57,58,60]	3849	187.51 (180.28, 194.75)	179.06 (171.94, 186.17)	161.97 (153.68, 170.26)	LR: *p* < 0.1 HR: *p* < 0.001	85.41%
Total Lymphocyte (10^3^/µL)	[30,33,38,39,41,44,46,49,50,52,53,55,57,58]	2423	1.96 (1.67, 2.25)	1.79 (1.50, 2.09)	1.56 (1.22, 1.89)	HR: *p* < 0.1	95.90%
Transferrin (mg/dL)	[23,27,28,30,41,46,50,52,53,56,58]	2988	273.29 (234.90, 311.68)	270.66 (223.50, 317.82)	258.96 (204.80, 313.12)	ns	98.83%
Prealbumin (mg/dL)	[23,30,32,39,41,43,46,48,53,56,59]	2823	24.19 (22.55, 25.83)	22.06 (20.36, 23.76)	19.13 (17.26, 21.00)	LR: *p* < 0.1 HR: *p* < 0.001	91.24%
C-reactive Protein (mg/L)	[23,32,34,41,43,47,48]	1714	39.22 (6.71, 71.72)	51.08 (18.43, 83.73)	55.16 (19.17, 91.14)	ns	99.60%
Creatinine (µmol/L)	[23,34,42,46,47,48,54,56,58]	2736	103.53 (96.09, 110.96)	105.45 (96.75, 114.15)	98.99 (89.83, 108.14)	ns	78.59%
Total Protein (g/dL)	[23,41,47,48,52,57,60]	1359	7.12 (6.67, 7.58)	6.97 (6.58, 7.37)	6.54 (6.10, 6.98)	HR: *p* < 0.05	94.63%
Triglycerides (mg/dL)	[23,28,34,39,43,47,48,54]	1972	129.74 (116.48, 142.99)	121.85 (108.21, 135.49)	118.27 (103.22, 133.32)	ns	81.12%
Iron (µg/dL)	[27,28,33,43,60]	1026	76.48 (61.21, 91.76)	63.14 (40.97, 85.31)	47.58 (13.79, 81.38)	HR: *p* < 0.1	90.58%
White Blood Cells (10^3^/µL)	[41,47,48,58]	1115	9.19 (5.56, 12.82)	9.22 (6.32, 12.12)	9.57 (6.61, 12.54)	ns	94.39%
Hematocrit (%)	[24,27,41,50,54]	592	40.59 (37.39, 43.79)	39.13 (34.75, 43.52)	36.80 (30.99, 42.61)	ns	92.15%
eGFR (mL/min/1.73 m^2^)	[32,34,43]	861	61.88 (48.34, 75.42)	57.30 (44.44, 70.15)	55.94 (40.88, 71.00)	ns	79.18%
Blood Urea Nitrogen (mmol/L)	[47,48,54,56]	1610	7.94 (6.86, 9.02)	8.09 (6.97, 9.20)	7.85 (6.50, 9.21)	ns	78.91%
Low-density Lipoprotein (mg/dL)	[32,34,47,54]	658	105.04 (94.85, 115.23)	99.08 (87.17, 110.99)	86.38 (69.40, 103.36)	HR: *p* < 0.05	46.23%
High-density Lipoprotein (mg/dL)	[34,43,54]	542	44.65 (30.40, 58.90)	43.32 (29.55, 57.09)	40.64 (26.51, 54.76)	ns	77.36%

^1^ Predicted mean for subjects of age 72 years old and 50% are female based on multivariate meta-regression analysis controlling for age and gender. Statistics (Stats) shows *p*-values by *t*-test comparing mean values of low risk (LR) or high risk (HR) group to that of no risk (NR). eGFR, estimated glomerular filtration rate; ns, non-significant.

**Table 2:** Mean biomarker levels in 3 subgroups of malnutrition risk status defined by MNA, included patients with acute disease ^1^.

Marker (Units)	References	*n*	MNA > 23.5 (No Risk) Mean (95% CI)	MNA 17-23.5 (Low Risk) Mean (95% CI)	MNA < 17 (High Risk) Mean (95% CI)	Stats	*I*^2^
BMI (kg/m^2^)	[24,30,32,33,34,35,38,39,40,41,42,43,44,45,46,47,48,49,50,51,52,53,54,55,61,62,63,64]	4886	27.30 (26.41, 28.19)	25.48 (24.58, 26.39)	20.05 (20.06, 22.04)	LR: *p* < 0.01 HR: *p* < 0.001	92.84%
Albumin (g/dL)	[23,24,28,30,32,33,34,35,38,39,40,41,43,45,46,47,48,49,50,51,52,55,56,57,58,59,60,61,62,63,64,65]	6930	3.80 (3.64, 3.95)	3.62 (3.47, 3.78)	3.32 (3.15, 3.49)	LR: *p* < 0.1 HR: *p* < 0.001	98.04%
Hemoglobin (g/dL)	[24,27,28,30,32,33,39,40,41,42,43,44,46,48,50,54,57,58,60,61,62]	4681	12.92 (12.53, 13.30)	12.45 (12.07, 12.84)	11.73 (11.29, 12.16)	LR: *p* < 0.1 HR: *p* < 0.001	93.57%
Total Cholesterol (mg/dL)	[23,28,30,34,38,39,41,43,46,47,48,50,52,54,57,58,60,61]	3980	187.49 (180.16, 194.83)	177.17 (170.15, 184.18)	161.49(153.47, 169.50)	LR: *p* < 0.05 HR: *p* < 0.001	85.93%
Total Lymphocyte (10^3^/µL)	[30,33,38,39,41,44,46,49,50,52,53,55,57,58,61,65]	2658	1.92 (1.66, 2.19)	1.81 (1.54, 2.07)	1.62 (1.33, 1.92)	ns	95.35%
Transferrin (mg/dL)	[23,27,28,30,41,46,50,52,53,56,58,62,64]	3077	271.49 (236.45, 306.53)	269.72 (227.22, 312.22)	252.54 (204.65, 300.42)	ns	98.43%
Prealbumin (mg/dL)	[23,30,32,39,41,43,46,48,53,56,59,65]	2927	23.83 (21.93, 25.73)	21.87 (19.89, 23.85)	19.30 (17.15, 21.45)	HR: *p* < 0.01	93.61%
C-reactive Protein (mg/L)	[23,32,34,41,43,47,48,62,64]	1803	35.98 (6.27, 65.68)	48.31 (17.12, 79.51)	53.48 (20.08, 86.87)	ns	99.56%
Creatinine (µmol/L)	[23,34,42,46,47,48,54,56,58,62]	2776	103.86 (96.65, 111.08)	105.55 (97.19, 113.92)	98.55 (89.72, 107.37)	ns	75.91%
Total Protein (g/dL)	[23,41,47,48,52,57,60,61,62]	1530	7.06 (6.70, 7.41)	6.93 (6.64, 7.22)	6.47 (6.17, 6.77)	HR: *p* < 0.01	91.92%
Triglycerides (mg/dL)	[23,28,34,39,43,47,48,54,61]	2103	129.73 (114.76, 144.71)	115.08 (100.76, 129.40)	111.90 (96.40, 127.40)	HR: *p* < 0.1	87.98%
Iron (µg/dL)	[27,28,33,43,60,62]	1066	76.91 (63.90, 89.93)	62.19 (43.85, 80.52)	49.96 (24.19, 75.72)	HR: *p* < 0.05	86.69%
White Blood Cells (10^3^/µL)	[41,47,48,58,61]	1246	8.69 (6.19, 11.18)	8.69 (6.77, 10.61)	8.99 (7.08, 10.90)	ns	92.39%
Hematocrit (%)	[24,27,41,50,54,62]	632	40.51 (36.92, 44.10)	41.11 (36.41, 45.81)	39.16 (33.13, 45.19)	ns	93.70%

^1^ Predicted mean for subjects of age 72 years old and 50% are female based on multivariate meta-regression analysis controlling for age and gender. Statistics (Stats) shows *p*-values by *t*-test comparing mean values of low risk (LR) or high risk (HR) group to that of no risk (NR). ns, non-significant.

**Table 3:** Mean biomarker levels in 3 subgroups of malnutrition risk status defined by SGA ^1^.

Marker (Units)	References	*n*	SGA A (No Risk) Mean (95% CI)	SGA B (Low Risk) Mean (95% CI)	SGA C (High Risk) Mean (95% CI)	Stats	*I*^2^
Non-Acute Patients Only							
BMI (kg/m^2^)	[26,49,66,67,68,69,70,71,72,73]	1946	26.16 (24.33, 27.99)	22.92 (21.23, 24.60)	19.77 (18.11, 21.42)	LR: *p* < 0.01 HR: *p* < 0.001	96.42%
Albumin (g/dL)	[26,49,59,66,67,68,69,70,71,72,73,74,75,76]	2765	3.68 (3.45, 3.92)	3.41 (3.18, 3.64)	3.08 (2.84, 3.31)	LR: *p* < 0.1 HR: *p* < 0.001	97.44%
Hemoglobin (g/dL)	[26,68,69,74,75,76]	917	12.84 (11.66, 14.02)	11.86 (10.71, 13.00)	10.34 (9.15, 11.52)	HR: *p* < 0.01	97.26%
Total Cholesterol (mg/dL)	[26,67,68,70,73,75]	925	202.73 (184.27, 221.19)	179.95 (163.77, 196.12)	166.24 (148.25, 184.23)	LR: *p* < 0.05 HR: *p* < 0.01	87.30%
Total Lymphocyte (10^3^/µL)	[26,49,66,69,75,76]	1434	1.95 (1.32, 2.58)	1.57 (1.00, 2.15)	1.37 (0.85, 1.89)	HR: *p* < 0.1	97.69%
Prealbumin (mg/dL)	[59,66,71,77]	1545	22.32 (15.88, 28.77)	18.07 (11.74, 24.39)	16.35 (9.56, 23.13)	ns	98.22%
C-reactive Protein (mg/L)	[66,67,69,73]	1279	119.27 (47.19, 191.34)	88.77 (31.44, 146.09)	91.97 (37.37, 146.58)	ns	95.26%
Total Protein (g/dL)	[26,67,68,69]	970	6.39 (5.36, 7.42)	5.85 (5.00, 6.71)	5.65 (4.93, 6.37)	LR: *p* < 0.1 HR: *p* < 0.05	91.84%
Included Acute Patients							
BMI (kg/m^2^)	[26,49,63,66,67,68,69,70,71,72,73,78,79,80]	2245	26.09 (24.47, 27.72)	22.67 (21.23, 24.11)	19.67 (18.27, 21.07)	LR: *p* < 0.001 HR: *p* < 0.001	95.97%
Albumin (g/dL)	[26,31,49,59,63,66,67,68,69,70,71,72,73,74,75,76,78,79,80,81]	3916	3.54 (3.31, 3.78)	3.30 (3.08, 3.52)	2.96 (2.74, 3.19)	HR: *p* < 0.001	97.95%
Hemoglobin (g/dL)	[26,68,69,74,75,76,78]	973	12.82 (11.74, 13.90)	11.88 (10.87, 12.89)	10.36 (9.33, 11.39)	HR: *p* < 0.001	96.59%
Total Lymphocyte (10^3^/µL)	[26,49,66,69,75,76,78]	1482	1.92 (1.27, 2.57)	1.45 (0.89, 2.01)	1.25 (0.74, 1.76)	HR: *p* < 0.05	97.80%
Prealbumin (mg/dL)	[59,66,71,77,78,81]	1712	20.61 (15.95, 25.27)	17.22 (12.98, 21.47)	14.63 (10.22, 19.04)	HR: *p* < 0.1	96.88%
C-reactive Protein (mg/L)	[66,67,69,73,78,79]	1451	110.32 (46.12, 174.52)	85.01 (34.06, 135.95)	92.50 (45.60, 139.40)	ns	93.87%
Total Protein (g/dL)	[26,67,68,69,79]	1094	6.32 (4.64, 8.00)	5.85 (4.44, 7.27)	5.41 (4.22, 6.61)	HR: *p* < 0.1	96.62%

^1^ Predicted mean for subjects of age 72 years old and 50% are female based on multivariate meta-regression analysis controlling for age and gender. Statistics (Stats) shows *p*-values by *t*-test comparing mean values of low risk (LR) or high risk (HR) group to that of no risk (NR). ns, non-significant.

**Table 4:** Mean biomarker levels in 2 subgroups of malnutrition risk status defined by NRS-2002 ^1^.

Marker (Units)	References	*n*	NRS-2002 < 3 (Low Risk) Mean (95% CI)	NRS-2002 ≥ 3 (High Risk) Mean (95% CI)	Stats	*I*^2^
Non-Acute Patients Only						
BMI (kg/m^2^)	[19,82,83,84,85,86,87]	17809	25.77 (23.90, 27.63)	22.60 (21.23, 23.97)	*p* < 0.01	98.12%
Albumin (g/dL)	[19,82,83,84,85,86,87,88]	17944	3.74 (3.44, 4.03)	3.42 (3.19, 3.64)	*p* < 0.1	98.62%
Hemoglobin (g/dL)	[82,83,85,87]	16686	13.24 (12.63, 13.86)	11.89 (11.48, 12.29)	*p* < 0.01	83.56%
Total Cholesterol (mg/dL)	[85,87,88]	15864	156.20 (42.64, 269.76)	132.61 (76.00, 189.22)	ns	97.45%
Total Lymphocyte (10^3^/µL)	[83,84,85,86,88]	2111	1.91 (1.58, 2.23)	1.62 (1.35, 1.90)	*p* < 0.1	87.94%
C-reactive Protein (mg/L)	[19,82,87]	15883	28.51 (−21.60, 78.62)	49.96 (18.05, 81.97)	ns	92.40%
Included Acute Patients						
BMI (kg/m^2^)	[19,82,83,84,85,86,87,89]	17954	26.40 (24.39, 28.42)	22.87 (21.38, 24.35)	*p* < 0.01	98.49%
Albumin (g/dL)	[19,65,82,83,84,85,86,87,88,90]	18577	3.56 (3.32, 3.81)	3.32 (3.12, 3.52)	*p* < 0.1	98.72%
Hemoglobin (g/dL)	[82,83,85,87,90]	17215	12.99 (12.50, 13.47)	11.87 (11.50, 12.25)	*p* < 0.01	83.98%
Total Cholesterol (mg/dL)	[85,87,88,89]	16009	172.56 (84.71, 260.41)	147.35 (108.22, 186.49)	ns	97.53%
Total Lymphocyte (10^3^/µL)	[65,83,84,85,86,88]	2215	1.70 (1.41, 2.00)	1.48 (1.25, 1.72)	ns	91.65%
C-reactive Protein (mg/L)	[19,82,87,90]	16362	53.48 (22.56, 84.41)	60.22 (34.70, 85.73)	ns	96.29%
Prealbumin (mg/dL)	[65,86,91]	706	18.09 (9.26, 26.92)	12.89 (3.98, 21.80)	ns	84.54%

^1^ Predicted mean for subjects of age 72 years old and 50% are female based on multivariate meta-regression analysis controlling for age and gender. Statistics (Stats) shows *p*-values by *t*-test comparing mean values of high risk (HR) group to that of low risk (LR). ns, non-significant.

**Table 5:** Mean biomarker levels in 3subgroups of malnutrition risk status defined by GNRI ^1^.

Marker (Units)	References	*n*	GNRI > 98 (No Risk) Mean (95% CI)	GNRI 92-98 (Low Risk) Mean (95% CI)	GNRI < 92 (High Risk) Mean (95% CI)	Stats	*I*^2^
Non-Acute Patients Only							
BMI (kg/m^2^)	[36,46,92,93,94,95]	4457	24.83 (23.64, 26.01)	23.96 (22.66, 25.25)	20.24 (18.46, 22.03)	LR: *p* < 0.05 HR: *p* < 0.001	73.26%
Albumin (g/dL)	[36,46,92,93,94,95]	4457	4.21 (4.12, 4.31)	3.75 (3.64, 3.85)	3.32 (3.18, 3.47)	LR: *p* < 0.001 HR: *p* < 0.001	85.67%
Total Lymphocyte (10^3^/µL)	[36,46,92]	755	1.69 (0.99, 2.38)	1.47 (0.81, 2.14)	1.23 (0.40, 2.07)	LR: *p* < 0.1 HR: *p* < 0.01	28.40%
Prealbumin (mg/dL)	[36,46,92,94]	3410	22.28 (18.46, 26.09)	20.15 (15.95, 24.34)	14.58 (9.18, 19.97)	LR: *p* < 0.05 HR: *p* < 0.001	42.37%
Included Acute Patients							
BMI (kg/m^2^)	[36,46,61,62,92,93,94,95]	4626	27.29 (26.16, 28.42)	26.11 (24.86, 27.35)	23.43 (21.86, 25.00)	LR: *p* < 0.1 HR: *p* < 0.001	87.11%
Albumin (g/dL)	[36,46,61,62,92,93,94,95]	4626	3.90 (3.73, 4.07)	3.44 (3.25, 3.62)	2.89 (2.66, 3.13)	LR: *p* < 0.001 HR: *p* < 0.001	98.23%
Hemoglobin (g/dL)	[46,61,62]	527	12.23 (10.66, 13.80)	12.19 (10.58, 13.80)	10.98 (9.28, 12.69)	ns	75.55%
Total Lymphocyte (10^3^/µL)	[36,46,61,92]	886	1.92 (1.63, 2.22)	1.82 (1.52, 2.13)	1.56 (1.20, 1.91)	HR: *p* < 0.05	74.47%
Transferrin (mg/dL)	[36,46,62]	616	203.00 (176.60, 229.39)	182.42 (159.21, 205.64)	169.05 (137.40, 200.69)	LR: *p* < 0.01 HR: *p* < 0.001	0.00%
Prealbumin (mg/dL)	[36,46,61,92,94]	3541	13.98 (11.55, 16.41)	10.96 (8.38, 13.53)	3.50 (0.56, 6.45)	LR: *p* < 0.05 HR: *p* < 0.001	85.53%

^1^ Predicted mean for subjects of age 72 years old and 50% are female based on multivariate meta-regression analysis controlling for age and gender. Statistics (Stats) shows *p*-values by *t*-test comparing mean values of low risk (LR) or high risk (HR) group to that of no risk (NR). ns, non-significant.

## Supplementary Materials

The following are available online at www.mdpi.com/2072-6643/9/8/829/s1, Figure S1: Forest plot of BMI by malnutrition risk groups classified by MNA; Figure S2: Forest plot of albumin by malnutrition risk groups classified by MNA; Figure S3: Forest plot of prealbumin by malnutrition risk groups classified by MNA, Figure S4: Forest plot of hemoglobin by malnutrition risk groups classified by MNA, Figure S5: Forest plot of total cholesterol by malnutrition risk groups classified by MNA; Figure S6: Funnel plot of albumin by malnutrition risk groups classified by MNA; Table S1: Literature search strategy by ProQuest Dialog service; Table S2: Results of quality assessment of included studies; Table S3: Mean biomarker levels in 2 subgroups of malnutrition risk status defined by MNA, excluded patients with acute disease; Table S4: Mean biomarker levels in 2 subgroups of malnutrition risk status defined by MNA, included patients with acute disease; Table S5: Mean biomarker levels in 2 subgroups of malnutrition risk status defined by SGA; Table S6: Mean biomarker levels in 2 subgroups of malnutrition risk status defined by MNA-SF; Table S7: Mean biomarker levels in 2 subgroups of malnutrition risk status defined by GNRI; Table S8: PRISMA checklist.
