# Supplementary material for: Evaluation of Blood Biomarkers Associated with Risk of Malnutrition in Older Adults: A Systematic Review and Meta-Analysis
Source: Nutrients. 2017 Aug 3;9(8):829. doi: 10.3390/nu9080829 (PMC5579622; doi:10.3390/nu9080829)

Author(s) and Year	N	%Female	Age	SD	Acute	Mean Alb [95% CI]
MNA <17						
Rohrig, 2015	130	68	81	7	N	3.44 [3.36, 3.52]
Abd−El−Gawad, 2014	53	36	70	9	Y	3.14 [2.94, 3.34]
Akin, 2014	18	78	82	7	N	3.90 [3.32, 4.48]
Kuyumcu, 2013	12	25	75	2	N	3.49 [3.07, 3.91]
Donini, 2013	36	75	84	9	N	3.43 [3.27, 3.59]
Rambousková, 2013	75	86	85	7	N	3.41 [3.34, 3.48]
Vischer (Case−control), 2012	29	63	85	6	N	2.75 [2.47, 3.03]
Vischer (Perpective), 2012	113	80	86	7	B	3.23 [3.13, 3.33]
Duran Alert, 2012	17	72	85	6	Y	3.00 [2.77, 3.23]
Alhamdan, 2011	31	65	71	9	N	2.72 [2.57, 2.87]
Bonilla−Palomas, 2011	27	80	79	8	N	3.50 [3.35, 3.65]
Cereda, 2011	116	75	86	8	N	3.49 [3.42, 3.56]
De Luis, 2011	137	79	85	7	N	3.60 [3.53, 3.67]
Calderon Reyes, 2010	47	59	70	8	N	2.98 [2.80, 3.16]
Prescha, 2010	14	50	49	15	N	3.65 [3.15, 4.15]
Drescher, 2010	23	78	84	8	Y	2.97 [2.81, 3.13]
Amirkalali, 2010	6	56	75	9	N	4.30 [4.08, 4.52]
Lei, 2009	36	53	68	6	N	3.47 [3.23, 3.71]
Venzin, 2009	44	48	66	16	B	3.76 [3.54, 3.98]
Inoue, 2007	45	71	80	9	N	3.50 [3.32, 3.68]
Reyes, 2007.2	18	58	71	8	N	2.70 [2.42, 2.98]
De Luis, 2006	48	56	77	15	N	2.80 [2.60, 3.00]
Kuzuya, 2005	45	64	80	8	N	3.60 [3.42, 3.78]
Kagansky, 2005.2	204	63	86	6	N	2.81 [2.74, 2.88]
Peña, 2004	10	51	82	5	N	3.50 [3.31, 3.69]
Gerber, 2003.2	12	100	89	6	N	3.96 [3.63, 4.29]
Magri, 2003	62	86	80	9	N	3.52 [3.38, 3.66]
Persson, 2002	18	68	79	9	Y	3.39 [3.10, 3.68]
Christensson, 2002.2	60	57	84	6	N	3.02 [2.88, 3.16]
Vellas (women+men), 2000	55	64	82	5	N	2.81 [2.65, 2.97]
Murphy, 2000	8	100	80	7	Y	3.38 [3.14, 3.62]
RE Model for Subgroup						3.32 [3.17, 3.46]
MNA 17−23.5						
Rohrig, 2015.1	378	68	81	7	N	3.61 [3.57, 3.65]
Abd−El−Gawad, 2014.1	69	54	69	8	Y	3.09 [2.96, 3.22]
Akin, 2014.1	33	73	83	8	N	4.00 [3.81, 4.19]
Kuyumcu, 2013.1	69	48	73	8	N	3.66 [3.52, 3.80]
Donini, 2013.1	46	67	82	9	N	3.66 [3.54, 3.78]
Rambousková, 2013.1	305	85	85	7	N	3.66 [3.63, 3.70]
Vischer (Case−control), 2012.1	82	63	85	6	N	3.07 [3.00, 3.14]
Vischer (Perpective), 2012.1	224	72	85	7	B	3.33 [3.24, 3.42]
Duran Alert, 2012.1	13	72	84	6	Y	3.42 [3.17, 3.67]
Alhamdan, 2011.1	43	65	71	9	N	3.08 [2.93, 3.23]
Bonilla−Palomas, 2011.1	127	48	73	10	N	3.80 [3.71, 3.89]
Cereda, 2011.1	206	76	84	8	N	3.64 [3.59, 3.69]
De Luis, 2011.1	245	63	83	9	N	3.80 [3.72, 3.88]
Calderon Reyes, 2010.1	86	59	70	8	N	3.95 [3.83, 4.07]
Prescha, 2010.1	80	49	49	15	N	4.00 [3.84, 4.16]
Drescher, 2010.1	50	78	84	8	Y	3.03 [2.93, 3.14]
Amirkalali, 2010.1	74	56	78	10	N	4.70 [4.60, 4.80]
Lei, 2009.1	98	35	68	6	N	3.97 [3.86, 4.08]
Venzin, 2009.1	85	48	61	21	B	3.91 [3.77, 4.05]
Inoue, 2007.1	83	64	79	9	N	3.70 [3.61, 3.79]
Reyes, 2007.1	49	58	71	8	N	3.00 [2.80, 3.20]
De Luis, 2006.1	107	47	74	15	N	3.30 [3.19, 3.41]
Kuzuya, 2005.1	131	69	78	7	N	4.10 [4.05, 4.15]
Soini (women+men), 2005	24	78	84	4	N	3.93 [3.76, 4.10]
Kagansky, 2005.1	137	66	84	6	N	3.13 [3.04, 3.22]
Delacorte, 2004.1	13	55	71	7	N	3.70 [3.37, 4.03]
Peña, 2004.1	44	51	77	7	N	3.60 [3.51, 3.69]
Gerber, 2003.1	45	100	86	6	N	4.27 [4.14, 4.40]
Magri, 2003.1	83	86	80	8	N	3.87 [3.78, 3.96]
Persson, 2002.1	38	68	83	6	Y	3.36 [3.22, 3.50]
Christensson, 2002.1	147	57	84	6	N	3.45 [3.37, 3.53]
Vellas (women+men), 2000.1	43	60	82	8	N	3.15 [3.01, 3.29]
Murphy, 2000.1	23	100	82	10	Y	3.62 [3.46, 3.78]
RE Model for Subgroup						3.62 [3.48, 3.76]
MNA >23.5						
Rohrig, 2015.2	104	68	81	7	N	3.75 [3.68, 3.83]
Abd−El−Gawad, 2014.2	9	89	68	8	Y	3.18 [2.75, 3.61]
Akin, 2014.2	103	65	81	5	N	4.30 [4.20, 4.40]
Kuyumcu, 2013.2	19	42	69	9	N	3.99 [3.74, 4.24]
Donini, 2013.2	18	72	74	10	N	3.83 [3.65, 4.01]
Rambousková, 2013.2	403	77	85	7	N	3.97 [3.94, 4.00]
Vischer (Case−control), 2012.2	52	63	86	7	N	3.21 [3.10, 3.32]
Vischer (Perpective), 2012.2	107	72	85	7	B	3.43 [3.34, 3.52]
Duran Alert, 2012.2	10	72	83	7	Y	3.48 [3.18, 3.78]
Alhamdan, 2011.2	11	65	71	9	N	3.30 [2.96, 3.64]
Bonilla−Palomas, 2011.2	57	23	71	11	N	3.80 [3.70, 3.90]
Cereda, 2011.2	36	71	81	9	N	3.76 [3.61, 3.91]
De Luis, 2011.2	111	50	83	9	N	3.90 [3.79, 4.01]
Calderon Reyes, 2010.2	24	59	70	8	N	4.22 [4.04, 4.40]
Prescha, 2010.2	81	38	49	15	N	4.16 [4.06, 4.26]
Drescher, 2010.2	31	78	84	8	Y	3.22 [3.07, 3.37]
Amirkalali, 2010.2	99	56	74	10	N	4.80 [4.71, 4.89]
Lei, 2009.2	50	34	68	6	N	4.09 [3.99, 4.19]
Venzin, 2009.2	294	48	63	19	B	4.13 [4.08, 4.18]
Inoue, 2007.2	53	64	81	8	N	4.00 [3.92, 4.08]
Reyes, 2007	30	58	71	8	N	3.30 [3.09, 3.51]
De Luis, 2006.2	45	42	67	15	N	3.41 [3.21, 3.61]
Kuzuya, 2005.2	50	78	77	7	N	4.40 [4.32, 4.48]
Soini (women+men), 2005.1	27	78	84	4	N	3.89 [3.75, 4.03]
Kagansky, 2005	72	71	84	5	N	3.31 [3.21, 3.41]
Delacorte, 2004	29	55	71	7	N	3.80 [3.51, 4.09]
Peña, 2004.2	9	51	72	7	N	3.40 [3.27, 3.53]
Gerber, 2003	21	100	85	7	N	4.57 [4.36, 4.78]
Magri, 2003.2	29	86	79	8	N	3.94 [3.72, 4.16]
Persson, 2002.2	12	68	87	5	Y	3.43 [3.15, 3.71]
Christensson, 2002	54	57	84	6	N	3.58 [3.46, 3.70]
Vellas (women+men), 2000.2	53	74	70	9	N	4.00 [3.92, 4.08]
Murphy, 2000.2	18	100	78	7	Y	3.93 [3.68, 4.18]
RE Model for Subgroup						3.81 [3.66, 3.96]
RE Model for All Studies						3.59 [3.50, 3.68]

Heterogeneity: $I^2 = 98\%$, $p < .0001$

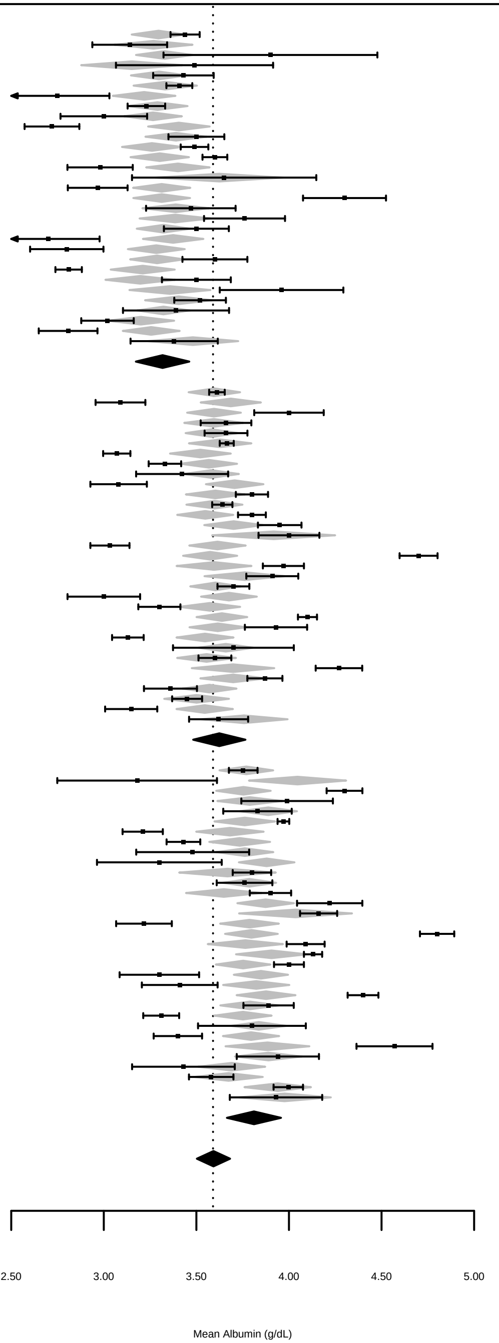

Supplement: Supplementary file 1 [file nutrients-09-00829-s001.zip › Figure S2-Forest-Alb-MNA.pdf]
