# Supplementary material for: Evaluation of Blood Biomarkers Associated with Risk of Malnutrition in Older Adults: A Systematic Review and Meta-Analysis
Source: Nutrients. 2017 Aug 3;9(8):829. doi: 10.3390/nu9080829 (PMC5579622; doi:10.3390/nu9080829)
Supplement: Supplementary file 1 [file nutrients-09-00829-s001.zip › Nutrients-211123-Supp-revised.pdf]

Supplementary Table S1. Literature search strategy by ProQuest Dialog service[1]

Set #	Keywords	Results
S5	(s1 and s2) not (s3)	686[3]
S4	Tio,ab,su,mesh,emb(animal or cat or feline or dog or canine or bird or avian or cow or bovine or heifer or Holstein or chick or chicken or insect or fly or rat or rats or rodent or mouse or mice or murine or chimp* or ape or hen or conference or "conference proceedings")	14394892[2]
S3	Tio,ab,su,mesh,emb("kidney disease" or "renal disease" or "renal failure" or "kidney failure" or tumour or tumor or oncology or cancer or neoplasms or hemodialysis or dialysis)	8792304[2]
S2	Tio,ab,su,mesh,emb(albumin or prealbumin or transthyretin or "total protein" or hemoglobin or "c-reactive protein" or "total lymphocyte count" or creatinine or leptin or "insulin-like growth factor-1" or "igf binding protein-1" or transferrin or "retinol-binding protein" or "total cholesterol" or triglyceride or "low-density lipoprotein cholesterol" or "high-density lipoprotein cholesterol" or orosomucoid or "25-hydroxycholecalciferol" or "red blood cell count" or "white blood cell count" or "blood urea nitrogen" or (homocysteine and "methylmalonic acid") or "pyridoxal-5-phosphate" or "gh-binding protein" or "a1-acid glycoprotein" or "tumor necrosis factor-alpha" or "interleukin-1 beta" or "interleukin-6" or "creatinine clearance" or "aspartate aminotransferase" or "alanine aminotransferase" or ferritin or cholinesterase or hematocrit)	2624944[2]
S1	Tio,ab,su,mesh,emb("mini nutritional assessment" or "subjective global assessment" or "malnutrition screening tool" or "malnutrition universal screening tool" or "nutritional risk screening" or "short nutritional assessment questionnaire" or "nutritional risk index" or "detailed nutritional assessment")	7475[2]

[1] Databases include Embase®, Embase® Alert, Foodline®: SCIENCE, FSTA®, MEDLINE®, ProQuest Dissertations and Theses Professional. [2] Duplicates are removed from the search, but included in the result count. [3] Duplicates are removed from the search and from the result count.

Supplementary Table S2. Results of quality assessment of included studies[1]

Year	Author	CK 1	CK 2	CK 3	CK 4	CK 5	CK 6	CK 7	CK 8	CK 9	CK 10	CK 11	CK 12	CK 13	CK 14	Quality
1995	Coppini	Y	Y	NR	Y	N	N	N	Y	Y	NA	Y	NR	NA	N	Fair
1997	Gloria	Y	Y	NR	Y	N	N	N	Y	Y	NA	Y	NR	NA	N	Fair
1998	de Groot	Y	Y	N	Y	N	N	N	N	Y	NA	Y	NR	NA	N	Fair
1999	Niyongabo	Y	Y	NR	Y	N	N	N	Y	Y	NA	Y	NR	NA	N	Fair
2000	Duerksen	Y	Y	NR	Y	N	N	N	Y	Y	NA	Y	Y	NA	N	Fair
2000	Murphy	Y	Y	NR	Y	N	N	N	Y	Y	NA	Y	NR	NA	N	Fair
2000	Sacks	Y	Y	Y	Y	N	N	N	Y	Y	NA	Y	Y	NA	N	Good
2000	Vellas	Y	Y	NR	N	N	N	N	Y	Y	NA	Y	NR	NA	N	Fair
2002	Christensson	Y	Y	Y	Y	N	N	N	Y	Y	NA	Y	NR	NA	N	Fair
2002	Covinsky	Y	Y	Y	Y	N	N	N	Y	Y	NA	Y	Y	NA	N	Good
2002	Persson	Y	Y	Y	Y	N	N	N	Y	Y	NA	Y	NR	NA	N	Fair
2003	Gerber	Y	Y	Y	Y	N	N	N	Y	Y	NA	Y	NR	NA	N	Fair
2003	Magri	Y	Y	NR	Y	N	N	N	Y	Y	NA	Y	NR	NA	N	Fair
2003	Pirlich	Y	Y	NR	Y	N	N	N	N	Y	NA	Y	NR	NA	N	Fair
2003	Ruiz-Lopez	Y	Y	Y	Y	N	N	N	Y	Y	NA	Y	NR	NA	N	Fair
2004	Delacorte	Y	Y	N	Y	N	N	N	N	Y	NA	Y	NR	NA	N	Fair
2004	Kyle	Y	Y	NR	Y	N	N	N	Y	Y	NA	Y	NR	NA	N	Fair
2004	Peña	Y	Y	NR	Y	N	N	N	Y	Y	NA	Y	NR	NA	N	Fair
2004	Sakarya	Y	Y	NR	Y	N	N	N	N	Y	NA	Y	NR	NA	N	Fair
2004	Sungurtekin (surgery)	Y	Y	NR	Y	N	N	N	Y	Y	NA	Y	NR	NA	N	Fair
2004	Sungurtekin	Y	Y	NR	Y	N	N	N	N	Y	NA	Y	NR	NA	N	Fair
2005	Alves de Rezende	Y	Y	Y	Y	N	N	N	Y	Y	NA	Y	NR	NA	N	Fair
2005	Bouillanne (3y)	Y	Y	NR	Y	N	N	N	Y	Y	NA	Y	NR	NA	N	Fair
2005	Bouillanne (6-mo)	Y	Y	NR	Y	N	N	N	Y	Y	NA	Y	NR	NA	N	Fair
2005	Gómez Ramos	Y	Y	NR	Y	N	N	N	N	Y	NA	Y	NR	NA	N	Fair
2005	Kagansky	Y	Y	Y	Y	N	N	N	Y	Y	NA	Y	NR	NA	N	Fair
2005	Kuzuya	Y	Y	NR	N	N	N	N	Y	Y	NA	Y	NR	NA	N	Fair

2005	Martineau	Y	Y	NR	Y	N	N	N	N	Y	NA	Y	NR	NA	N	Poor
2005	Sağıroğlu	Y	Y	NR	Y	N	N	N	Y	Y	NA	Y	NR	NA	N	Fair
2005	Soini	Y	Y	Y	Y	N	N	N	Y	Y	NA	Y	NR	NA	N	Fair
2005	Valero	Y	Y	NR	Y	N	N	N	N	Y	NA	Y	NR	NA	N	Fair
2006	Bonin-Guillaume	Y	Y	NR	Y	N	N	N	Y	Y	NA	Y	NR	NA	N	Fair
2006	Cereda	Y	Y	NR	Y	N	N	N	Y	Y	NA	Y	NR	NA	N	Fair
2006	De Luis	Y	Y	NR	Y	Y	N	N	Y	Y	NA	Y	NR	NA	N	Fair
2006	Kruizenga	Y	Y	NR	Y	N	N	N	N	Y	NA	Y	NR	NA	N	Fair
2007	Inoue	Y	Y	NR	Y	N	N	N	Y	Y	NA	Y	NR	NA	N	Fair
2007	Neumann	Y	Y	Y	Y	N	N	N	N	Y	NA	Y	Y	NA	N	Fair
2007	Pham	Y	Y	NR	Y	N	N	N	Y	Y	NA	Y	NR	NA	N	Fair
2007	Reyes	Y	Y	NR	Y	N	N	N	Y	Y	NA	Y	NR	NA	N	Fair
2008	Atalay	Y	Y	NR	Y	N	N	N	Y	Y	NA	Y	NR	NA	N	Poor
2008	Cereda	Y	Y	NR	Y	N	N	N	Y	Y	NA	Y	NR	NA	N	Fair
2008	Cereda (PIMAI)	Y	Y	NR	Y	N	N	N	Y	Y	NA	Y	NR	NA	N	Fair
2008	Salvi	Y	Y	NR	Y	N	N	N	N	Y	NA	Y	NR	NA	N	Fair
2008	Sungurtekin	Y	Y	NR	Y	N	N	N	Y	Y	NA	Y	NR	NA	N	Fair
2009	Lei	Y	Y	NR	Y	N	N	N	Y	Y	NA	Y	NR	NA	N	Fair
2009	Venzin	Y	Y	Y	Y	N	N	N	Y	Y	NA	Y	NR	NA	N	Fair
2010	Amirkalali	Y	Y	NR	Y	N	N	N	Y	Y	NA	Y	NR	NA	N	Fair
2010	Calderon Reyes	Y	Y	NR	Y	Y	N	N	Y	Y	NA	Y	NR	NA	N	Fair
2010	Cereda	Y	Y	N	Y	N	N	N	N	Y	NA	Y	NR	NA	N	Poor
2010	Drescher	Y	Y	Y	Y	N	N	N	Y	Y	NA	Y	NR	NA	N	Fair
2010	Filipovic	Y	Y	NR	Y	N	N	N	N	Y	NA	Y	NR	NA	N	Fair
2010	Gupta	Y	Y	NR	Y	N	N	N	Y	Y	NA	Y	NR	NA	N	Fair
2010	Prescha	Y	Y	NR	Y	N	N	N	Y	Y	NA	Y	NR	NA	N	Fair
2010	Saka	Y	Y	NR	Y	N	N	N	N	Y	NA	Y	NR	NA	N	Fair
2010	Sánchez-Muñoz	Y	Y	NR	Y	N	N	N	N	Y	NA	Y	NR	NA	N	Fair
2010	Ulger	Y	Y	NR	Y	N	N	N	N	Y	NA	Y	NR	NA	N	Fair
2011	Alhamdan	Y	Y	NR	Y	N	N	N	Y	Y	NA	Y	NR	NA	N	Fair

2011	Bonilla-Palomas	Y	Y	NR	Y	N	N	N	Y	Y	NA	Y	NR	NA	N	Fair
2011	Cereda	Y	Y	Y	Y	N	N	N	Y	Y	NA	Y	NR	NA	N	Fair
2011	De Luis	Y	Y	NR	Y	Y	N	N	Y	Y	NA	Y	NR	NA	N	Fair
2011	Filipovic	Y	Y	NR	Y	N	N	N	Y	Y	NA	Y	NR	NA	N	Fair
2011	Kaburagi	Y	Y	NR	Y	N	N	N	N	Y	NA	Y	NR	NA	N	Fair
2011	Kim	Y	Y	Y	Y	N	N	N	Y	Y	NA	Y	NR	NA	N	Fair
2011	Leandro-Merhi	Y	Y	NR	Y	N	N	N	Y	Y	NA	Y	NR	NA	N	Fair
2011	Mirarefin	Y	Y	NR	Y	N	N	N	N	Y	NA	Y	NR	NA	N	Fair
2012	Al-Najjar	Y	Y	NR	Y	N	N	N	N	Y	NA	Y	NR	NA	N	Poor
2012	Duran Alert	Y	Y	NR	Y	N	N	N	Y	Y	NA	Y	NR	NA	N	Fair
2012	Gallo	Y	Y	Y	Y	N	N	N	Y	Y	NA	Y	NR	NA	N	Fair
2012	Ji	Y	Y	Y	Y	N	N	N	Y	Y	NA	Y	NR	NA	N	Fair
2012	Nogay	Y	Y	NR	Y	N	N	N	Y	Y	NA	Y	NR	NA	N	Fair
2012	Vischer (Case-control)	Y	Y	NR	Y	N	N	N	Y	Y	NA	Y	NR	NA	N	Fair
2012	Vischer (Perpective)	Y	Y	NR	Y	N	N	N	Y	Y	NA	Y	NR	NA	N	Fair
2013	Boban	Y	Y	NR	Y	N	N	N	N	Y	NA	Y	NR	NA	N	Fair
2013	Donini	Y	Y	Y	Y	N	N	N	Y	Y	NA	Y	NR	NA	N	Fair
2013	Guo	Y	Y	NR	Y	N	N	N	N	Y	NA	Y	NR	NA	N	Fair
2013	Holst	Y	Y	Y	Y	N	N	N	N	Y	NA	Y	NR	NA	N	Fair
2013	Kinugasa	Y	Y	Y	Y	N	N	N	N	Y	NA	Y	NR	NA	N	Poor
2013	Kuyumcu	Y	Y	NR	Y	N	N	N	Y	Y	NA	Y	NR	NA	N	Fair
2013	Lee	Y	Y	NR	Y	N	N	N	N	Y	NA	Y	NR	NA	N	Fair
2013	Nykanen	Y	Y	Y	Y	N	N	N	N	Y	NA	Y	NR	NA	N	Fair
2013	Olveira	Y	Y	NR	Y	N	N	N	Y	Y	NA	Y	NR	NA	N	Fair
2013	Rambousková	Y	Y	NR	Y	N	N	N	Y	Y	NA	Y	NR	NA	N	Fair
2013	Sargento	Y	Y	NR	Y	N	N	N	N	Y	NA	Y	NR	NA	N	Fair
2013	Soysal	Y	Y	NR	Y	N	N	N	Y	Y	NA	Y	NR	NA	N	Fair
2014	Abd-El-Gawad	Y	Y	Y	Y	N	N	N	Y	Y	NA	Y	NR	NA	N	Fair
2014	Akin	Y	Y	NR	Y	N	N	N	Y	Y	NA	Y	NR	NA	N	Fair
2014	Bassim	Y	Y	NR	Y	N	N	N	N	Y	NA	Y	NR	NA	N	Fair

2014	Drevet	Y	Y	NR	Y	N	N	N	N	Y	NA	Y	NR	NA	N	Fair
2014	Mayasari	Y	Y	NR	Y	Y	N	N	N	Y	NA	Y	NR	NA	N	Fair
2014	Rasheed	Y	Y	Y	Y	N	N	N	Y	Y	NA	Y	NR	NA	N	Fair
2014	Trufa	Y	Y	NR	Y	N	N	N	N	Y	NA	Y	NR	NA	N	Fair
2014	Yosry	Y	Y	NR	Y	N	N	N	N	Y	NA	Y	NR	NA	N	Fair
2015	Demir	Y	Y	NR	Y	N	N	N	Y	Y	NA	Y	NR	NA	N	Fair
2015	Jeejeebhoy	Y	Y	Y	Y	Y	N	N	Y	Y	NA	Y	NR	NA	N	Good
2015	Kissova	Y	Y	Y	Y	N	N	N	N	Y	NA	Y	NR	NA	N	Poor
2015	Konturek	Y	Y	NR	Y	N	N	N	N	Y	NA	Y	NR	NA	N	Fair
2015	Matsumura	Y	Y	NR	Y	N	N	N	N	Y	NA	Y	NR	NA	N	Fair
2015	Rohrig	Y	Y	Y	Y	N	N	N	Y	Y	NA	Y	NR	NA	N	Poor
2015	Zhou	Y	Y	NR	Y	N	N	N	Y	Y	N	Y	Y	NA	N	Fair
2016	Alzahrani	Y	Y	N	Y	N	N	N	N	Y	N	Y	NR	NA	N	Fair
2016	Savita	Y	Y	NR	Y	N	N	N	Y	Y	N	Y	NR	NA	N	Fair
2016	Budzyński	Y	Y	Y	Y	N	N	N	N	Y	N	Y	NR	NA	N	Fair
2016	Felder	Y	Y	NR	Y	N	N	N	Y	Y	N	Y	NR	NA	N	Fair
2016	Gartner	Y	Y	NR	Y	N	N	N	Y	Y	N	Y	NR	NA	N	Fair
2016	Inoue	Y	Y	Y	Y	N	N	N	Y	Y	N	Y	NR	NA	N	Fair
2016	Izawa	Y	Y	NR	Y	N	N	N	N	Y	N	Y	NR	NA	N	Fair
2016	Rossi	Y	Y	Y	Y	N	N	N	N	Y	N	Y	NR	NA	N	Fair
2016	Shakersain	Y	Y	Y	Y	N	N	N	Y	Y	N	Y	NR	NA	N	Fair
2017	Hosseini	Y	Y	NR	Y	N	N	N	Y	Y	N	Y	NR	NA	N	Fair
2017	Kunimura	Y	Y	NR	Y	N	N	N	Y	Y	N	Y	NR	NA	N	Fair
2017	Yoo	Y	Y	Y	Y	N	N	N	N	Y	N	Y	NR	NA	N	Poor

[1] Quality of included studies was assessed using the National Institutes of Health (NIH) Quality Assessment tool for Observational Cohort and Cross-Sectional Studies (https://www.nhlbi.nih.gov/health-pro/guidelines/in-develop/cardiovascular-risk-reduction/tools/cohort). CK 1. Was the research question or objective in this paper clearly stated? CK 2. Was the study population clearly specified and defined? CK 3. Was the participation rate of eligible persons at least 50%? CK 4. Were all the subjects selected or recruited from the same or similar populations (including the same time period)? Were inclusion and exclusion criteria for being in the study prespecified and applied uniformly to all participants? CK 5. Was a sample size justification, power description, or variance and effect estimates provided? CK 6. For the analyses in this paper, were the exposure(s) of interest measured prior to the outcome(s) being measured? CK 7. Was the timeframe sufficient so that one could

reasonably expect to see an association between exposure and outcome if it existed? CK 8. For exposures that can vary in amount or level, did the study examine different levels of the exposure as related to the outcome (e.g., categories of exposure, or exposure measured as continuous variable)? CK 9. Were the exposure measures (independent variables) clearly defined, valid, reliable, and implemented consistently across all study participants? CK 10. Was the exposure(s) assessed more than once over time? CK 11. Were the outcome measures (dependent variables) clearly defined, valid, reliable, and implemented consistently across all study participants? CK 12. Were the outcome assessors blinded to the exposure status of participants? CK 13. Was loss to follow-up after baseline 20% or less? CK 14. Were key potential confounding variables measured and adjusted statistically for their impact on the relationship between exposure(s) and outcome(s)? CK, check list; CD, cannot be determined; NA, not applicable; NR, not reported; N, no; Y, yes.

Supplementary Table S3. Mean biomarker levels in 2 subgroups of malnutrition risk status defined by MNA, excluded patients with acute disease[1]

			MNA > 23.5 (No Risk)	MNA ≤ 23.5 (At Risk)	Stats	I^2
**Marker (Units)**	**References**	**n**	**Mean (95% CI)**	**Mean (95% CI)**		
BMI (kg/m^2)	[1-29]	6141	27.03 (26.17, 27.90)	23.91 (22.99, 24.84)	P<0.001	94.37%
Albumin (g/dL)	[1-4,6,8-19,21-28,30-39]	8629	3.89 (3.74, 4.05)	3.63 (3.46, 3.80)	p<0.01	99.43%
Hemoglobin (g/dL)	[1,3-7,9,10,12,13,17,21,22,24,25,29,31-33,36,37,39,40]	5981	13.12 (12.73, 13.50)	12.52 (12.12, 12.92)	p<0.05	96.83%
Total Cholesterol (mg/dL)	[2,4,6,9-13,16,17,19,25,26,29,31-33,35,37,39]	4797	189.31 (180.05, 198.57)	178.86 (169.40, 188.32)	p<0.1	95.25%
Total Lymphocyte ($10^3/\mu L$)	[4,7,9,10,12,15-17,19-21,25,26,28,32,33,38]	2874	1.97 (1.72, 2.21)	1.76 (1.50, 2.02)	ns	96.29%
Transferrin (mg/dL)	[4,9,10,17,19,20,26,30,31,33,35,40]	3094	269.45 (231.04, 307.85)	263.34 (213.99, 312.69)	ns	99.07%
Prealbumin (mg/dL)	[3,4,6,9,10,12,13,20,26,30,34,35]	2929	23.97 (22.37, 25.57)	21.32 (19.61, 23.03)	p<0.05	92.70%
C-reactive Protein (mg/L)	[2-4,6,11,13,23,35,36]	2360	39.80 (9.56, 70.03)	54.59 (24.18, 84.99)	ns	99.69%
Creatinine (μmol/L)	[2,5,10,11,13,29,30,33,35]	2736	103.95 (96.76, 111.14)	105.60 (97.21, 114.00)	ns	80.82%
Total Protein (g/dL)	[4,11,13,19,24-26,32,35,36,39]	2243	6.96 (6.60, 7.32)	6.75 (6.41, 7.10)	ns	97.36%
Triglycerides (mg/dL)	[2,6,11-13,29,31,35]	1972	131.27 (117.91, 144.64)	124.79 (111.45, 138.13)	ns	83.10%
Iron (μg/dL)	[6,21,31,39,40]	1026	77.26 (56.13, 98.40)	61.31 (20.68, 101.94)	ns	95.44%
White Blood Cells ($10^3/\mu L$)	[4,11,13,25,33,37]	1940	8.12 (5.47, 10.77)	8.39 (5.96, 10.82)	ns	98.46%
Hematocrit (%)	[4,17,22,29,37,40]	1292	41.30 (38.11, 44.50)	39.96 (35.22, 44.70)	ns	96.25%
eGFR (mL/min/1.73 m^2)	[2,3,6]	861	58.94 (50.64, 67.24)	56.84 (48.80, 64.88)	ns	6.52%
Blood Urea Nitrogen (mmol/L)	[11,13,29,30]	1610	7.94 (6.58, 9.30)	8.12 (6.73, 9.52)	ns	86.92%
Low-density Lipoprotein (mg/dL)	[2,3,11,29]	788	107.87 (101.35, 114.38)	105.50 (98.06, 112.95)	ns	15.06%
High-density Lipoprotein (mg/dL)	[2,6,25,29]	672	50.20 (38.67, 61.74)	49.83 (37.31, 62.35)	ns	86.98%

[1] Predicted mean for subjects of age 72 years old and 50% are female based on multivariate meta-regression analysis controlling for age and gender. Statistics (Stats) shows p-values by t-test comparing mean values of at risk (AR) group to that of no risk (NR). eGFR, estimated glomerular filtration rate; ns, non-significant.

Supplementary Table S4. Mean biomarker levels in 2 subgroups of malnutrition risk status defined by MNA, included patients with acute disease[1]

			MNA > 23.5 (No Risk)	MNA ≤ 23.5 (At Risk)	Stats	I^2
**Marker (Units)**	**References**	**n**	**Mean (95% CI)**	**Mean (95% CI)**		
BMI (kg/m^2)	[1-29,41-45]	6429	27.13 (26.32, 27.95)	24.08 (23.23, 24.93)	p<0.001	93.57%
Albumin (g/dL)	[1-4,6,8-19,21-28,30-39,41-46]	9323	3.86 (3.70, 4.01)	3.61 (3.45, 3.77)	p<0.01	99.40%
Hemoglobin (g/dL)	[1,3-7,9,10,12,13,17,21,22,24,25,29,31-33,36,37,39,40,42,45]	6454	13.03 (12.65, 13.41)	12.45 (12.06, 12.84)	p<0.05	96.81%
Total Cholesterol (mg/dL)	[2,4,6,9-13,16,17,19,25,26,29,31-33,35,37,39,41]	4928	189.00 (179.80, 198.20)	177.20 (168.11, 186.29)	p<0.1	95.15%
Total Lymphocyte (10^3/µL)	[4,7,9,10,12,15-17,19-21,25,26,28,32,33,38,41,46]	3109	1.93 (1.70, 2.16)	1.78 (1.54, 2.01)	ns	95.93%
Transferrin (mg/dL)	[4,9,10,17,19,20,26,30,31,33,35,40,42,44]	3183	268.06 (233.05, 303.06)	262.14 (217.84, 306.45)	ns	98.77%
Prealbumin (mg/dL)	[3,4,6,9,10,12,13,20,26,30,34,35,46]	3033	23.71 (21.81, 25.62)	21.29 (19.24, 23.34)	p<0.05	94.96%
C-reactive Protein (mg/L)	[2-4,6,11,13,23,35,36,42,44,45]	2751	34.13 (9.46, 58.81)	49.42 (23.07, 75.78)	ns	99.61%
Creatinine (µmol/L)	[2,5,10,11,13,29,30,33,35,42]	2776	104.31 (97.15, 111.46)	105.29 (96.98, 113.60)	ns	78.45%
Total Protein (g/dL)	[4,11,13,19,24-26,32,35,36,39,41,42]	2414	6.95 (6.62, 7.27)	6.75 (6.46, 7.03)	ns	96.68%
Triglycerides (mg/dL)	[2,6,11-13,29,31,35,41]	2103	131.88 (116.11, 147.64)	118.32 (103.52, 133.12)	ns	90.33%
Iron (µg/dL)	[6,21,31,39,40,42]	1066	77.54 (60.29, 94.79)	59.09 (29.31, 88.88)	ns	93.32%
White Blood Cells (10^3/µL)	[4,11,13,25,33,37,41]	2071	8.03 (5.81, 10.26)	8.25 (6.43, 10.08)	ns	97.83%
Hematocrit (%)	[4,17,22,29,37,40,42,45]	1634	41.80 (38.40, 45.20)	42.17 (37.55, 46.78)	ns	97.25%

[1] Predicted mean for subjects of age 72 years old and 50% are female based on multivariate meta-regression analysis controlling for age and gender. Statistics (Stats) shows p-values by t-test comparing mean values of at risk (AR) group to that of no risk (NR). ns, non-significant.

Supplementary Table S5. Mean biomarker levels in 2 subgroups of malnutrition risk status defined by SGA[1]

			SGA A (No Risk)	SGA B/C (At Risk)	Stats	I^2
**Marker (units)**	**References**	**n**	**Mean (95% CI)**	**Mean (95% CI)**		
Non-acute Patients Only						
BMI (kg/m^2)	[15,47-59]	2915	26.48 (24.80, 28.16)	21.81 (20.39, 23.22)	p<0.001	95.60%
Albumin (g/dL)	[15,34,47-63]	4685	3.70 (3.48, 3.92)	3.33 (3.12, 3.53)	p<0.01	98.25%
Hemoglobin (g/dL)	[47,50,51,60-63]	1666	12.80 (11.42, 14.18)	11.32 (10.10, 12.55)	p<0.1	97.92%
Total Cholesterol (mg/dL)	[47,49,50,52,56,59,61]	1466	196.66 (176.33, 216.98)	168.09 (151.62, 184.56)	p<0.01	92.38%
Total Lymphocyte (10^3/µL)	[15,47,48,51,56,59,61,62]	1691	1.54 (0.86, 2.12)	1.18 (0.63, 1.74)	ns	97.57%
Prealbumin (mg/dL)	[34,48,53,64,65]	1588	21.68 (15.82, 27.54)	16.49 (10.65, 22.33)	ns	98.37%
C-reactive Protein (mg/L)	[48,49,51,56,59]	1530	104.04 (21.60, 186.48)	82.01 (21.84, 142.18)	ns	94.25%
Total Protein (g/dL)	[47,49-51]	970	6.54 (5.14, 7.93)	5.83 (4.80, 6.87)	p<0.1	93.69%
Included Acute Patients						
BMI (kg/m^2)	[15,43,47-59,66-70]	3497	26.50 (25.26, 27.75)	21.68 (20.62, 22.74)	p<0.001	94.64%
Albumin (g/dL)	[15,34,43,47-63,66-72]	5909	3.62 (3.39, 3.84)	3.25 (3.05, 3.45)	p<0.01	98.69%
Hemoglobin (g/dL)	[47,50,51,60-63,67]	1714	12.81 (11.51, 14.12)	11.27 (10.14, 12.40)	p<0.05	97.63%
Total Lymphocyte (10^3/µL)	[15,47,48,51,56,59,61,62,67]	1739	1.52 (0.84, 2.21)	1.09 (0.55, 1.63)	ns	97.72%
Prealbumin (mg/dL)	[34,48,53,64,65,67,72]	1755	20.71 (16.05, 25.36)	15.95 (11.60, 20.30)	ns	97.75%
C-reactive Protein (mg/L)	[48,49,51,56,59,67,68]	1702	100.73 (32.35, 169.11)	84.36 (34.40, 134.32)	ns	92.84%
Total Protein (g/dL)	[47,49-51,68]	1094	6.53 (4.74, 8.32)	5.79 (4.44, 7.15)	p<0.1	96.46%

[1] Predicted mean for subjects of age 72 years old and 50% are female based on multivariate meta-regression analysis controlling for age and gender. Statistics (Stats) shows p-values by t-test comparing mean values of at risk (AR) group to that of no risk (NR). ns, non-significant.

Supplementary Table S6. Mean biomarker levels in 2 subgroups of malnutrition risk status defined by MNA-SF[1]

			MNA-SF > 11 (No Risk)	MNA-SF ≤ 11 (At Risk)	Stats	I^2
**Marker (Units)**	**References**	**n**	**Mean (95% CI)**	**Mean (95% CI)**		
Non-acute Patients Only						
BMI (kg/m^2)	[5,73-78]	5380	26.79 (24.39, 29.20)	23.49 (20.94, 26.04)	p<0.05	99.09%
Albumin (g/dL)	[73-80]	7869	3.89 (3.58, 4.20)	3.66 (3.32, 4.00)	ns	99.76%
Hemoglobin (g/dL)	[5,73-75,77-80]	6672	13.11 (12.54, 13.67)	12.24 (11.59, 12.89)	p<0.05	98.76%
Total Lymphocyte ($10^3/\mu L$)	[41,73,75,80]	556	1.81 (0.47, 3.15)	1.62 (0.22, 3.21)	ns	86.72%
Creatinine (μmol/L)	[5,76,79]	2992	103.47 (38.71, 168.23)	133.89 (-126.78, 394.56)	ns	89.22%
Included Acute Patients						
BMI (kg/m^2)	[5,41,43,73-78,81-83]	6025	27.65 (25.84, 29.46)	24.50 (22.64, 26.36)	p<0.01	98.74%
Albumin (g/dL)	[41,43,73-83]	8696	3.79 (3.55, 4.03)	3.62 (3.37, 3.87)	ns	99.60%
Hemoglobin (g/dL)	[5,41,73-75,77-80,82]	7078	13.02 (12.53, 13.52)	12.15 (11.61, 12.70)	p<0.05	98.58%
Total Cholesterol (mg/dL)	[41,76,79,82]	3348	189.23 (169.01, 209.45)	163.77 (141.75, 185.78)	p<0.1	94.33%
Total Lymphocyte ($10^3/\mu L$)	[41,73,75,80]	687	1.82 (1.51, 2.11)	1.62 (1.29, 1.96)	ns	71.34%
Creatinine (μmol/L)	[5,76,79,82]	3267	105.70 (83.70, 127.70)	137.70 (86.82, 188.59)	ns	86.38%

[1] Predicted mean for subjects of age 72 years old and 50% are female based on multivariate meta-regression analysis controlling for age and gender. Statistics (Stats) shows p-values by t-test comparing mean values of at risk (AR) group to that of no risk (NR). ns, non-significant.

Supplementary Table S7. Mean biomarker levels in 2 subgroups of malnutrition risk status defined by GNRI[1]

			GNRI > 98 (No Risk)	GNRI ≤ 98 (At Risk)	Stats[b]	I^2
**Marker (Units)**	**References**	**n**	**Mean (95% CI)**	**Mean (95% CI)**		
Non-acute Patients Only						
BMI (kg/m^2)	[10,84-89]	4995	25.54 (23.44, 27.64)	22.50 (19.73, 25.28)	p<0.001	93.56%
Albumin (g/dL)	[10,84-89]	4995	4.15 (3.94, 4.36)	3.53 (3.26, 3.81)	p<0.001	96.98%
Total Lymphocyte (10^3/μL)	[10,84,85]	755	1.80 (1.35, 2.25)	1.50 (1.04, 1.96)	p<0.01	0.00%
Prealbumin (mg/dL)	[10,84,85,87]	3410	24.46 (19.84, 29.07)	20.31 (14.60, 26.02)	p<0.01	58.06%
Included Acute Patients						
BMI (kg/m^2)	[10,41,42,84-89]	5164	28.06 (26.52, 29.61)	25.05 (23.06, 27.04)	p<0.01	95.66%
Albumin (g/dL)	[10,41,42,84-89]	5164	3.85 (3.64, 4.07)	3.15 (2.87, 3.42)	p<0.001	98.99%
Hemoglobin (g/dL)	[10,41,42,88]	1065	12.29 (11.37, 13.22)	11.34 (10.14, 12.55)	ns	82.78%
Total Lymphocyte (10^3/μL)	[10,41,84,85]	886	1.87 (1.56, 2.19)	1.65 (1.32, 1.99)	p<0.1	60.81%
Transferrin (mg/dL)	[10,42,85]	616	213.98 (177.33, 250.63)	187.83 (150.33, 225.34)	p<0.01	0.00%
Prealbumin (mg/dL)	[10,41,84,85,87]	3541	13.45 (9.74, 17.16)	6.84 (2.89, 10.79)	p<0.01	92.82%

[1] Predicted mean for subjects of age 72 years old and 50% are female based on multivariate meta-regression analysis controlling for age and gender. Statistics (Stats) shows p-values by t-test comparing mean values of at risk (AR) group to that of no risk (NR). ns, non-significant.

Supplementary Table S8. PRISMA checklist

Section/topic	#	Checklist item	Reported on page #
TITLE			
Title	1	Identify the report as a systematic review, meta-analysis, or both.	1
ABSTRACT			
Structured summary	2	Provide a structured summary including, as applicable: background; objectives; data sources; study eligibility criteria, participants, and interventions; study appraisal and synthesis methods; results; limitations; conclusions and implications of key findings; systematic review registration number.	1
INTRODUCTION			
Rationale	3	Describe the rationale for the review in the context of what is already known.	1, 2
Objectives	4	Provide an explicit statement of questions being addressed with reference to participants, interventions, comparisons, outcomes, and study design (PICOS).	2
METHODS			
Protocol and registration	5	Indicate if a review protocol exists, if and where it can be accessed (e.g., Web address), and, if available, provide registration information including registration number.	NA
Eligibility criteria	6	Specify study characteristics (e.g., PICOS, length of follow-up) and report characteristics (e.g., years considered, language, publication status) used as criteria for eligibility, giving rationale.	2, 3
Information sources	7	Describe all information sources (e.g., databases with dates of coverage, contact with study authors to identify additional studies) in the search and date last searched.	2, 3
Search	8	Present full electronic search strategy for at least one database, including any limits used, such that it could be repeated.	3, Table S6
Study selection	9	State the process for selecting studies (i.e., screening, eligibility, included in systematic review, and, if applicable, included in the meta-analysis).	3, Fig 1.
Data collection process	10	Describe method of data extraction from reports (e.g., piloted forms, independently, in duplicate) and any processes for obtaining and confirming data from investigators.	3

Data items	11	List and define all variables for which data were sought (e.g., PICOS, funding sources) and any assumptions and simplifications made.	3
Risk of bias in individual studies	12	Describe methods used for assessing risk of bias of individual studies (including specification of whether this was done at the study or outcome level), and how this information is to be used in any data synthesis.	3
Summary measures	13	State the principal summary measures (e.g., risk ratio, difference in means).	3
Synthesis of results	14	Describe the methods of handling data and combining results of studies, if done, including measures of consistency (e.g., I^2) for each meta-analysis.	3, 4
Risk of bias across studies	15	Specify any assessment of risk of bias that may affect the cumulative evidence (e.g., publication bias, selective reporting within studies).	3
Additional analyses	16	Describe methods of additional analyses (e.g., sensitivity or subgroup analyses, meta-regression), if done, indicating which were pre-specified.	4
RESULTS			
Study selection	17	Give numbers of studies screened, assessed for eligibility, and included in the review, with reasons for exclusions at each stage, ideally with a flow diagram.	4, Fig. 1
Study characteristics	18	For each study, present characteristics for which data were extracted (e.g., study size, PICOS, follow-up period) and provide the citations.	4, Table 1-5
Risk of bias within studies	19	Present data on risk of bias of each study and, if available, any outcome level assessment (see item 12).	Table S8
Results of individual studies	20	For all outcomes considered (benefits or harms), present, for each study: (a) simple summary data for each intervention group (b) effect estimates and confidence intervals, ideally with a forest plot.	Table 1-5, Fig. S1-4
Synthesis of results	21	Present results of each meta-analysis done, including confidence intervals and measures of consistency.	5, Table 1-5,
Risk of bias across studies	22	Present results of any assessment of risk of bias across studies (see Item 15).	6, Figure S5
Additional analysis	23	Give results of additional analyses, if done (e.g., sensitivity or subgroup analyses, meta-regression [see Item 16]).	6, 7
DISCUSSION			
Summary of evidence	24	Summarize the main findings including the strength of evidence for each main outcome; consider their relevance to key groups (e.g., healthcare providers, users, and policy makers).	13, 14
Limitations	25	Discuss limitations at study and outcome level (e.g., risk of bias), and at review-level (e.g., incomplete retrieval of identified research, reporting bias).	14, 15

Conclusions	26	Provide a general interpretation of the results in the context of other evidence, and implications for future research.	15
FUNDING			
Funding	27	Describe sources of funding for the systematic review and other support (e.g., supply of data); role of funders for the systematic review.	15

From: Moher D, Liberati A, Tetzlaff J, Altman DG, The PRISMA Group (2009). Preferred Reporting Items for Systematic Reviews and Meta-Analyses: The PRISMA Statement. PLoS Med 6(7): e1000097. doi:10.1371/journal.pmed1000097

For more information, visit: **www.prisma-statement.org**.

References

1. Rohrig, G.; Becker, I.; Polidori, M.C.; Schulz, R.J.; Noreik, M. Association of anemia and hypoalbuminemia in german geriatric inpatients: Relationship to nutritional status and comprehensive geriatric assessment. *Zeitschrift fur Gerontologie und Geriatrie* **2015**, *48*, 619-624.
2. Akin, S.; Tufan, F.; Bulut, L.; Genc, S.; Bahat, G.; Saka, B.; Erten, N.; Karan, M.A. Limited utility of adipokine levels in the diagnosis of malnutrition in the elderly. *Aging clinical and experimental research* **2014**, *26*, 229-234.
3. Kuyumcu, M.E.; Yeşil, Y.; Oztürk, Z.A.; Halil, M.; Ulger, Z.; Yavuz, B.B.; Cankurtaran, M.; Güngör, E.; Erdoğan, G.; Besler, T.*, et al.* Challenges in nutritional evaluation of hospitalized elderly; always with mini-nutritional assessment? *European Geriatric Medicine* **2013**, *4*, 231-236.
4. Donini, L.M.; Neri, B.; De Chiara, S.; Poggiogalle, E.; Muscaritoli, M. Nutritional care in a nursing home in italy. *PLoS One* **2013**, *8*, e55804.
5. Sargento, L.; Satendra, M.; Almeida, I.; Sousa, C.; Gomes, S.; Salazar, F.; Lousada, N.; Palma Dos Reis, R. Nutritional status of geriatric outpatients with systolic heart failure and its prognostic value regarding death or hospitalization, biomarkers and quality of life. *The journal of nutrition, health & aging* **2013**, *17*, 300-304.
6. Vischer, U.M.; Frangos, E.; Graf, C.; Gold, G.; Weiss, L.; Herrmann, F.R.; Zekry, D. The prognostic significance of malnutrition as assessed by the mini nutritional assessment (mna) in older hospitalized patients with a heavy disease burden. *Clinical nutrition (Edinburgh, Scotland)* **2012**, *31*, 113-117.
7. Leandro-Merhi, V.A.; De Aquino, J.L. Anthropometric parameters of nutritional assessment as predictive factors of the mini nutritional assessment (mna) of hospitalized elderly patients. *The journal of nutrition, health & aging* **2011**, *15*, 181-186.
8. Alhamdan, A.A.; Alsaif, A.A. The nutritional, glutathione and oxidant status of elderly subjects admitted to a university hospital. *Saudi journal of gastroenterology : official journal of the Saudi Gastroenterology Association* **2011**, *17*, 58-63.
9. Bonilla-Palomas, J.L.; Gamez-Lopez, A.L.; Anguita-Sanchez, M.P.; Castillo-Dominguez, J.C.; Garcia-Fuertes, D.; Crespin-Crespin, M.; Lopez-Granados, A.; Suarez de Lezo, J. [impact of malnutrition on long-term mortality in hospitalized patients with heart failure]. *Revista espanola de cardiologia* **2011**, *64*, 752-758.
10. Cereda, E.; Pedrolli, C.; Zagami, A.; Vanotti, A.; Piffer, S.; Opizzi, A.; Rondanelli, M.; Caccialanza, R. Nutritional screening and mortality in newly institutionalised elderly: A comparison between the geriatric nutritional risk index and the mini nutritional assessment. *Clinical nutrition (Edinburgh, Scotland)* **2011**, *30*, 793-798.
11. Amirkalali, B.; Sharifi, F.; Fakhrzadeh, H.; Mirarefein, M.; Ghaderpanahi, M.; Badamchizadeh, Z.; Larijani, B. Low serum leptin serves as a biomarker of malnutrition in elderly patients. *Nutrition research (New York, N.Y.)* **2010**, *30*, 314-319.
12. Lei, Z.; Qingyi, D.; Feng, G.; Chen, W.; Hock, R.S.; Changli, W. Clinical study of mini-nutritional assessment for older chinese inpatients. *The journal of nutrition, health & aging* **2009**, *13*, 871-875.
13. Venzin, R.M.; Kamber, N.; Keller, W.C.; Suter, P.M.; Reinhart, W.H. How important is malnutrition? A prospective study in internal medicine. *European journal of clinical nutrition* **2009**, *63*, 430-436.
14. Inoue, K.; Kato, M. Usefulness of the mini-nutritional assessment (mna) to evaluate the nutritional status of japanese frail elderly under home care. *Geriatrics & gerontology international* **2007**, *7*, 238-244.
15. Reyes, J.G.; Zuniga, A.S.; Cruz, M.G. [prevalence of hyponutrition in the elderly at admission to the hospital]. *Nutricion hospitalaria* **2007**, *22*, 702-709.
16. Kuzuya, M.; Kanda, S.; Koike, T.; Suzuki, Y.; Satake, S.; Iguchi, A. Evaluation of mini-nutritional assessment for japanese frail elderly. *Nutrition (Burbank, Los Angeles County, Calif.)* **2005**, *21*, 498-503.
17. Peña, E.; Meertens de R, L.; Solano, L. Valoración antropométrica y bioquímica de ancianos venezolanos institucionalizados. *Revista Española de Geriatría y Gerontología* **2004**, *39*, 360-366.
18. Gerber, V.; Krieg, M.A.; Cornuz, J.; Guigoz, Y.; Burckhardt, P. Nutritional status using the mini nutritional assessment questionnaire and its relationship with bone quality in a population of institutionalized elderly women. *The journal of nutrition, health & aging* **2003**, *7*, 140-145.
19. Magri, F.; Borza, A.; del Vecchio, S.; Chytiris, S.; Cuzzoni, G.; Busconi, L.; Rebesco, A.; Ferrari, E. Nutritional assessment of demented patients: A descriptive study. *Aging clinical and experimental research* **2003**, *15*, 148-153.
20. Ruiz-Lopez, M.D.; Artacho, R.; Oliva, P.; Moreno-Torres, R.; Bolanos, J.; de Teresa, C.; Lopez, M.C. Nutritional risk in institutionalized older women determined by the mini nutritional assessment test: What are the main factors? *Nutrition (Burbank, Los Angeles County, Calif.)* **2003**, *19*, 767-771.
21. Delacorte, R.R.; Moriguti, J.C.; Matos, F.D.; Pfrimer, K.; Marchinil, J.S.; Ferriolli, E. Mini-nutritional assessment score and the risk for undernutrition in free-living older persons. *The journal of nutrition, health & aging* **2004**, *8*, 531-534.
22. Soini, H.; Routasalo, P.; Lagstrom, H. Nutritional status in cognitively intact older people receiving home care services--a pilot study. *The journal of nutrition, health & aging* **2005**, *9*, 249-253.
23. Holst, M.; Yifter-Lindgren, E.; Surowiak, M.; Nielsen, K.; Mowe, M.; Carlsson, M.; Jacobsen, B.; Cederholm, T.; Fenger-Groen, M.; Rasmussen, H. Nutritional screening and risk factors in elderly hospitalized patients: Association to clinical outcome? *Scandinavian journal of caring sciences* **2013**, *27*, 953-961.

24. Mirarefin, M.; Sharifi, F.; Fakhrzadeh, H.; Nazari, N.; Ghaderpanahi, M.; Badamchizade, Z.; Tajalizadekhoob, Y. Predicting the value of the mini nutritional assessment (mna) as an indicator of functional ability in older iranian adults (kahrizak elderly study). *The journal of nutrition, health & aging* **2011**, *15*, 175-180.
25. Kaburagi, T.; Hirasawa, R.; Yoshino, H.; Odaka, Y.; Satomi, M.; Nakano, M.; Fujimoto, E.; Kabasawa, K.; Sato, K. Nutritional status is strongly correlated with grip strength and depression in community-living elderly japanese. *Public health nutrition* **2011**, *14*, 1893-1899.
26. Sánchez-Muñoz, L.A.; Calvo-Reyes, M.C.; Majo-Carbajo, Y.; Barbado-Ajo, J.; Aragón De La Fuente, M.M.; Artero-Ruiz, E.C.; Municio-Saldaña, M.I.; Jimeno-Carruez, A. Cribado nutricional con mini nutritional assessment (mna) en medicina interna. Ventajas e inconvenientes. *Revista Clínica Española* **2010**, *210*, 429-437.
27. Neumann, S.A.; Miller, M.D.; Daniels, L.A.; Ahern, M.; Crotty, M. Mini nutritional assessment in geriatric rehabilitation: Inter-rater reliability and relationship to body composition and nutritional biochemistry. *Nutrition & Dietetics* **2007**, *64*, 179-185.
28. Sakarya, M.; Karadag, F.; Luleci, N.; Tezcan Keles, G.; Topcu, I.; Erincler, T. [relationship between nutrition and asa-classification in the elderly]. *Anasthesiologie, Intensivmedizin, Notfallmedizin, Schmerztherapie : AINS* **2004**, *39*, 400-405.
29. Hosseini, S.; Keshavarz, S.A.; Amin, A.; Bakshandeh, H.; Maleki, M.; Shahinfard, A.; Hosseini, S.; Heidarali, M. Nutritional status assessment of the elderly patients with congestive heart failure by mini nutritional assessment test. *Res Cardiovasc Med* **2017**, *6*, e31898.
30. Rambouskova, J.; Slavikova, M.; Krskova, A.; Prochazka, B.; Andel, M.; Dlouhy, P. Nutritional status assessment of institutionalized elderly in prague, czech republic. *Annals of nutrition & metabolism* **2013**, *62*, 201-206.
31. De Luis, D.A.; Lopez Mongil, R.; Gonzalez Sagrado, M.; Lopez Trigo, J.A.; Mora, P.F.; Castrodeza Sanz, J. Nutritional status in a multicenter study among institutionalized patients in spain. *European review for medical and pharmacological sciences* **2011**, *15*, 259-265.
32. Calderon Reyes, M.E.; Ibarra Ramirez, F.; Garcia, J.; Gomez Alonso, C.; Rodriguez-Orozco, A.R. [compared nutritional assessment for older adults at family medicine settings]. *Nutricion hospitalaria* **2010**, *25*, 669-675.
33. Kagansky, N.; Berner, Y.; Koren-Morag, N.; Perelman, L.; Knobler, H.; Levy, S. Poor nutritional habits are predictors of poor outcome in very old hospitalized patients. *The American journal of clinical nutrition* **2005**, *82*, 784-791; quiz 913-784.
34. Christensson, L.; Unosson, M.; Ek, A.C. Evaluation of nutritional assessment techniques in elderly people newly admitted to municipal care. *European journal of clinical nutrition* **2002**, *56*, 810-818.
35. Vellas, B.; Guigoz, Y.; Baumgartner, M.; Garry, P.J.; Lauque, S.; Albarede, J.L. Relationships between nutritional markers and the mini-nutritional assessment in 155 older persons. *J Am Geriatr Soc* **2000**, *48*, 1300-1309.
36. Saka, B.; Kaya, O.; Ozturk, G.B.; Erten, N.; Karan, M.A. Malnutrition in the elderly and its relationship with other geriatric syndromes. *Clinical nutrition (Edinburgh, Scotland)* **2010**, *29*, 745-748.
37. de Groot, L.C.; Beck, A.M.; Schroll, M.; van Staveren, W.A. Evaluating the determine your nutritional health checklist and the mini nutritional assessment as tools to identify nutritional problems in elderly europeans. *European journal of clinical nutrition* **1998**, *52*, 877-883.
38. de Luis, D.; Lopez Guzman, A.; Nutrition Group of Society of, C.-L. Nutritional status of adult patients admitted to internal medicine departments in public hospitals in castilla y leon, spain - a multi-center study. *European journal of internal medicine* **2006**, *17*, 556-560.
39. Prescha, A.; Pieczyñska, J.; Biernat, K.; Neubauer, K.; Smereka, A.; Ilow, R.; Grajeta, H.; Biernat, J.; Paradowski, L. Nutritional status assessment of patients with inflammatory bowel disease. *Ocena stanu odżywienia pacjentów z nieswoistym zapaleniem jelit.* **2010**, *17*, 25-31.
40. Alves de Rezende, C.H.; Marquez Cunha, T.; Alvarenga Junior, V.; Penha-Silva, N. Dependence of mini-nutritional assessment scores with age and some hematological variables in elderly institutionalized patients. *Gerontology* **2005**, *51*, 316-321.
41. Abd-El-Gawad, W.M.; Abou-Hashem, R.M.; El Maraghy, M.O.; Amin, G.E. The validity of geriatric nutrition risk index: Simple tool for prediction of nutritional-related complication of hospitalized elderly patients. Comparison with mini nutritional assessment. *Clinical nutrition (Edinburgh, Scotland)* **2014**, *33*, 1108-1116.
42. Duran Alert, P.; Mila Villarroel, R.; Formiga, F.; Virgili Casas, N.; Vilarasau Farre, C. Assessing risk screening methods of malnutrition in geriatric patients: Mini nutritional assessment (mna) versus geriatric nutritional risk index (gnri). *Nutricion hospitalaria* **2012**, *27*, 590-598.
43. Persson, M.D.; Brismar, K.E.; Katzarski, K.S.; Nordenstrom, J.; Cederholm, T.E. Nutritional status using mini nutritional assessment and subjective global assessment predict mortality in geriatric patients. *J Am Geriatr Soc* **2002**, *50*, 1996-2002.
44. Murphy, M.C.; Brooks, C.N.; New, S.A.; Lumbers, M.L. The use of the mini-nutritional assessment (mna) tool in elderly orthopaedic patients. *European journal of clinical nutrition* **2000**, *54*, 555-562.
45. Rossi, A.P.; Zanandrea, V.; Zoico, E.; Zanardo, M.; Caliari, C.; Confente, S.; Gabriele, S.; Mazzali, G.; Fantin, F.; Zamboni, M. Inflammation and nutritional status as predictors of physical performance and strength loss during hospitalization. *European journal of clinical nutrition* **2016**, *70*, 1439-1442.
46. Drescher, T.; Singler, K.; Ulrich, A.; Koller, M.; Keller, U.; Christ-Crain, M.; Kressig, R.W. Comparison of two malnutrition risk screening methods (mna and nrs 2002) and their association with markers of protein malnutrition in geriatric hospitalized patients. *European journal of clinical nutrition* **2010**, *64*, 887-893.

47. Demir, M.V.; Tamer, A.; Cinemre, H.; Uslan, I.; Yaylaci, S.; Erkorkmaz, U. Nutritional status and laboratory parameters among internal medicine inpatients. *Nigerian journal of clinical practice* **2015**, *18*, 757-761.
48. Olveira, G.; Tapia, M.J.; Ocón, J.; Cabrejas-Gómez, C.; Ballesteros-Pomar, M.D.; Vidal-Casariego, A.; Arraiza-Irigoyen, C.; Olivares, J.; Conde-García, M.C.; García-Manzanares, Á.*, et al.* The subjective global assessment predicts in-hospital mortality better than other nutrition-related risk indexes in noncritically ill inpatients who receive total parenteral nutrition in spain (prospective multicenter study). *Journal of the Academy of Nutrition and Dietetics* **2013**, *113*, 1209-1218.
49. Filipovic, B.; Kovcevic, N.; Randjelovic, T.; Kostic, S.; Filipovic, B. Nutritional status in hospitalized patients in the department of gastroenterohepatology. *Hepato-gastroenterology* **2011**, *58*, 1229-1234.
50. Gupta, B.; Kant, S.; Mishra, R. Subjective global assessment of nutritional status of chronic obstructive pulmonary disease patients on admission. *The international journal of tuberculosis and lung disease : the official journal of the International Union against Tuberculosis and Lung Disease* **2010**, *14*, 500-505.
51. Pham, N.V.; Cox-Reijven, P.L.; Wodzig, W.K.; Greve, J.W.; Soeters, P.B. Sga and measures for muscle mass and strength in surgical vietnamese patients. *Nutrition (Burbank, Los Angeles County, Calif.)* **2007**, *23*, 283-291.
52. Sacks, G.S.; Dearman, K.; Replogle, W.H.; Cora, V.L.; Meeks, M.; Canada, T. Use of subjective global assessment to identify nutrition-associated complications and death in geriatric long-term care facility residents. *Journal of the American College of Nutrition* **2000**, *19*, 570-577.
53. Niyongabo, T.; Melchior, J.C.; Henzel, D.; Bouchaud, O.; Larouze, B. Comparison of methods for assessing nutritional status in hiv-infected adults. *Nutrition (Burbank, Los Angeles County, Calif.)* **1999**, *15*, 740-743.
54. Gloria, L.; Cravo, M.; Camilo, M.E.; Resende, M.; Cardoso, J.N.; Oliveira, A.G.; Leitao, C.N.; Mira, F.C. Nutritional deficiencies in chronic alcoholics: Relation to dietary intake and alcohol consumption. *The American journal of gastroenterology* **1997**, *92*, 485-489.
55. Kissova, V.; Rosenberger, J.; Goboova, M.; Kiss, A. Ten-year all-cause mortality in hospitalized non-surgical patients based on nutritional status screening. *Public health nutrition* **2015**, *18*, 2609-2614.
56. Sungurtekin, H.; Sungurtekin, U.; Hanci, V.; Erdem, E. Comparison of two nutrition assessment techniques in hospitalized patients. *Nutrition (Burbank, Los Angeles County, Calif.)* **2004**, *20*, 428-432.
57. Pirlich, M.; Schutz, T.; Kemps, M.; Luhman, N.; Burmester, G.R.; Baumann, G.; Plauth, M.; Lubke, H.J.; Lochs, H. Prevalence of malnutrition in hospitalized medical patients: Impact of underlying disease. *Digestive diseases* **2003**, *21*, 245-251.
58. Scolapio, J.S.; Bowen, J.; Stoner, G.; Tarrosa, V. Substrate oxidation in patients with cirrhosis: Comparison with other nutritional markers. *JPEN. Journal of parenteral and enteral nutrition* **2000**, *24*, 150-153.
59. Sungurtekin, H.; Sungurtekin, U.; Balci, C.; Zencir, M.; Erdem, E. The influence of nutritional status on complications after major intraabdominal surgery. *Journal of the American College of Nutrition* **2004**, *23*, 227-232.
60. Nogay, N.H. The relationship of subjective global assessment with respiratory function and other nutrition parameters in copd. *HEALTHMED* **2012**, *6*, 2013-2017.
61. Duerksen, D.R.; Yeo, T.A.; Siemens, J.L.; O'Connor, M.P. The validity and reproducibility of clinical assessment of nutritional status in the elderly. *Nutrition (Burbank, Los Angeles County, Calif.)* **2000**, *16*, 740-744.
62. Coppini, L.Z.; Waitzberg, D.L.; Ferrini, M.T.; da Silva, M.L.; Gama-Rodrigues, J.; Ciosak, S.L. [comparison of the subjective global nutrition assessment x objective nutrition evaluation]. *Revista da Associacao Medica Brasileira* **1995**, *41*, 6-10.
63. Sağıroğlu, A.E.; Koltka, N.; Öztekin, F.; Yeşil, H.; Kaşıkçı, T. To estimate the nutritional status of the surgical patients with two different tecniques. *Med Med J* **2005**, *20*, 129-133.
64. Gallo, F.; Lucarini, S.; Boicelli, R.; Marchello, C.; Bravo, M.F.; Cavagnaro, P. Sorveglianza nutrizionale e prevenzione della malnutrizione nella popolazione istituzionalizzata della asl4 chiavarese. *GIORNALE DI GERONTOLOGIA* **2012**, *60*, 264-271.
65. Trufa, D.I.; Arhire, L.I.; Nita, O.; Gherasim, A.; Nita, G.; Graur, M. The evaluation of preoperative nutritional status in patients undergoing thoracic surgery. *Revista medico-chirurgicala a Societatii de Medici si Naturalisti din Iasi* **2014**, *118*, 514-519.
66. Bassim, C.W.; Fassil, H.; Dobbin, M.; Steinberg, S.M.; Baird, K.; Cole, K.; Joe, G.; Comis, L.E.; Mitchell, S.A.; Grkovic, L.*, et al.* Malnutrition in patients with chronic gvhd. *Bone marrow transplantation* **2014**, *49*, 1300-1306.
67. Kim, H.; Choi-Kwon, S. Changes in nutritional status in icu patients receiving enteral tube feeding: A prospective descriptive study. *Intensive & critical care nursing : the official journal of the British Association of Critical Care Nurses* **2011**, *27*, 194-201.
68. Sungurtekin, H.; Sungurtekin, U.; Oner, O.; Okke, D. Nutrition assessment in critically ill patients. *Nutrition in clinical practice : official publication of the American Society for Parenteral and Enteral Nutrition* **2008**, *23*, 635-641.
69. Martineau, J.; Bauer, J.D.; Isenring, E.; Cohen, S. Malnutrition determined by the patient-generated subjective global assessment is associated with poor outcomes in acute stroke patients. *Clinical nutrition (Edinburgh, Scotland)* **2005**, *24*, 1073-1077.
70. Bector, S.; Vagianos, K.; Suh, M.; Duerksen, D.R. Does the subjective global assessment predict outcome in critically ill medical patients? *Journal of Intensive Care Medicine* **2016**, *31*, 485-489.
71. Jeejeebhoy, K.N.; Keller, H.; Gramlich, L.; Allard, J.P.; Laporte, M.; Duerksen, D.R.; Payette, H.; Bernier, P.; Vesnaver, E.; Davidson, B.*, et al.* Nutritional assessment: Comparison of clinical assessment and objective

variables for the prediction of length of hospital stay and readmission. *The American journal of clinical nutrition* **2015**, *101*, 956-965.
72. Atalay, B.G.; Yagmur, C.; Nursal, T.Z.; Atalay, H.; Noyan, T. Use of subjective global assessment and clinical outcomes in critically ill geriatric patients receiving nutrition support. *JPEN. Journal of parenteral and enteral nutrition* **2008**, *32*, 454-459.
73. Zhou, J.; Wang, M.; Wang, H.; Chi, Q. Comparison of two nutrition assessment tools in surgical elderly inpatients in northern china. *Nutrition journal* **2015**, *14*, 68.
74. Shakersain, B.; Santoni, G.; Faxen-Irving, G.; Rizzuto, D.; Fratiglioni, L.; Xu, W. Nutritional status and survival among old adults: An 11-year population-based longitudinal study. *European journal of clinical nutrition* **2015**.
75. Mayasari, M.; Lestariana, W. Simple nutritional screening tool (snst) has good validity to identify risk of malnutrition on hospitalized elderly patients. *Pakistan Journal of Nutrition* **2014**, *13*, 573.
76. Soysal, P.; Isik, A.T.; Ugur, A.; Kazancioglu, R.; Ergun, F.; Babacan Yildiz, G. Vitamin b12 and folic acid levels are not related to length of stay in elderly inpatients. *Nutrition (Burbank, Los Angeles County, Calif.)* **2013**, *29*, 757-759.
77. Nykanen, I.; Lonnroos, E.; Kautiainen, H.; Sulkava, R.; Hartikainen, S. Nutritional screening in a population-based cohort of community-dwelling older people. *European journal of public health* **2013**, *23*, 405-409.
78. Ji, L.; Meng, H.; Dong, B. Factors associated with poor nutritional status among the oldest-old. *Clinical nutrition (Edinburgh, Scotland)* **2012**, *31*, 922-926.
79. Ulger, Z.; Halil, M.; Kalan, I.; Yavuz, B.B.; Cankurtaran, M.; Gungor, E.; Ariogul, S. Comprehensive assessment of malnutrition risk and related factors in a large group of community-dwelling older adults. *Clinical nutrition (Edinburgh, Scotland)* **2010**, *29*, 507-511.
80. Alzahrani, S.H.; El Sayed, I.A.; Alshamrani, S.M. Prevalence and factors associated with geriatric malnutrition in an outpatient clinic of a teaching hospital in jeddah, saudi arabia. *Ann Saudi Med* **2016**, *36*, 346-351.
81. Rasheed, S.; Woods, R.T. An investigation into the association between nutritional status and quality of life in older people admitted to hospital. *Journal of human nutrition and dietetics : the official journal of the British Dietetic Association* **2014**, *27*, 142-151.
82. Salvi, F.; Giorgi, R.; Grilli, A.; Morichi, V.; Espinosa, E.; Spazzafumo, L.; Marinozzi, M.L.; Dessi-Fulgheri, P. Mini nutritional assessment (short form) and functional decline in older patients admitted to an acute medical ward. *Aging clinical and experimental research* **2008**, *20*, 322-328.
83. Inoue, T.; Misu, S.; Tanaka, T.; Sakamoto, H.; Iwata, K.; Chuman, Y.; Ono, R. Pre-fracture nutritional status is predictive of functional status at discharge during the acute phase with hip fracture patients: A multicenter prospective cohort study. *Clinical Nutrition* **2016**.
84. Cereda, E.; Limonta, D.; Pusani, C.; Vanotti, A. Assessing elderly at risk of malnutrition: The new geriatric nutritional risk index versus nutritional risk index. *Nutrition (Burbank, Los Angeles County, Calif.)* **2006**, *22*, 680-682.
85. Cereda, E.; Pusani, C.; Limonta, D.; Vanotti, A. The association of geriatric nutritional risk index and total lymphocyte count with short-term nutrition-related complications in institutionalised elderly. *Journal of the American College of Nutrition* **2008**, *27*, 406-413.
86. Cereda, E.; Zagami, A.; Vanotti, A.; Piffer, S.; Pedrolli, C. Geriatric nutritional risk index and overall-cause mortality prediction in institutionalised elderly: A 3-year survival analysis. *Clinical nutrition (Edinburgh, Scotland)* **2008**, *27*, 717-723.
87. Bouillanne, O.; Morineau, G.; Dupont, C.; Coulombel, I.; Vincent, J.P.; Nicolis, I.; Benazeth, S.; Cynober, L.; Aussel, C. Geriatric nutritional risk index: A new index for evaluating at-risk elderly medical patients. *The American journal of clinical nutrition* **2005**, *82*, 777-783.
88. Al-Najjar, Y.; Clark, A.L. Predicting outcome in patients with left ventricular systolic chronic heart failure using a nutritional risk index. *The American journal of cardiology* **2012**, *109*, 1315-1320.
89. Kunimura, A.; Ishii, H.; Uetani, T.; Aoki, T.; Harada, K.; Hirayama, K.; Negishi, Y.; Shibata, Y.; Sumi, T.; Kawashima, K.*, et al.* Impact of geriatric nutritional risk index on cardiovascular outcomes in patients with stable coronary artery disease. *Journal of Cardiology* **2017**, *69*, 383-388.
