# Supplementary figures and images for: Evaluation of Blood Biomarkers Associated with Risk of Malnutrition in Older Adults: A Systematic Review and Meta-Analysis
Source: Nutrients. 2017 Aug 3;9(8):829. doi: 10.3390/nu9080829 (PMC5579622; doi:10.3390/nu9080829)

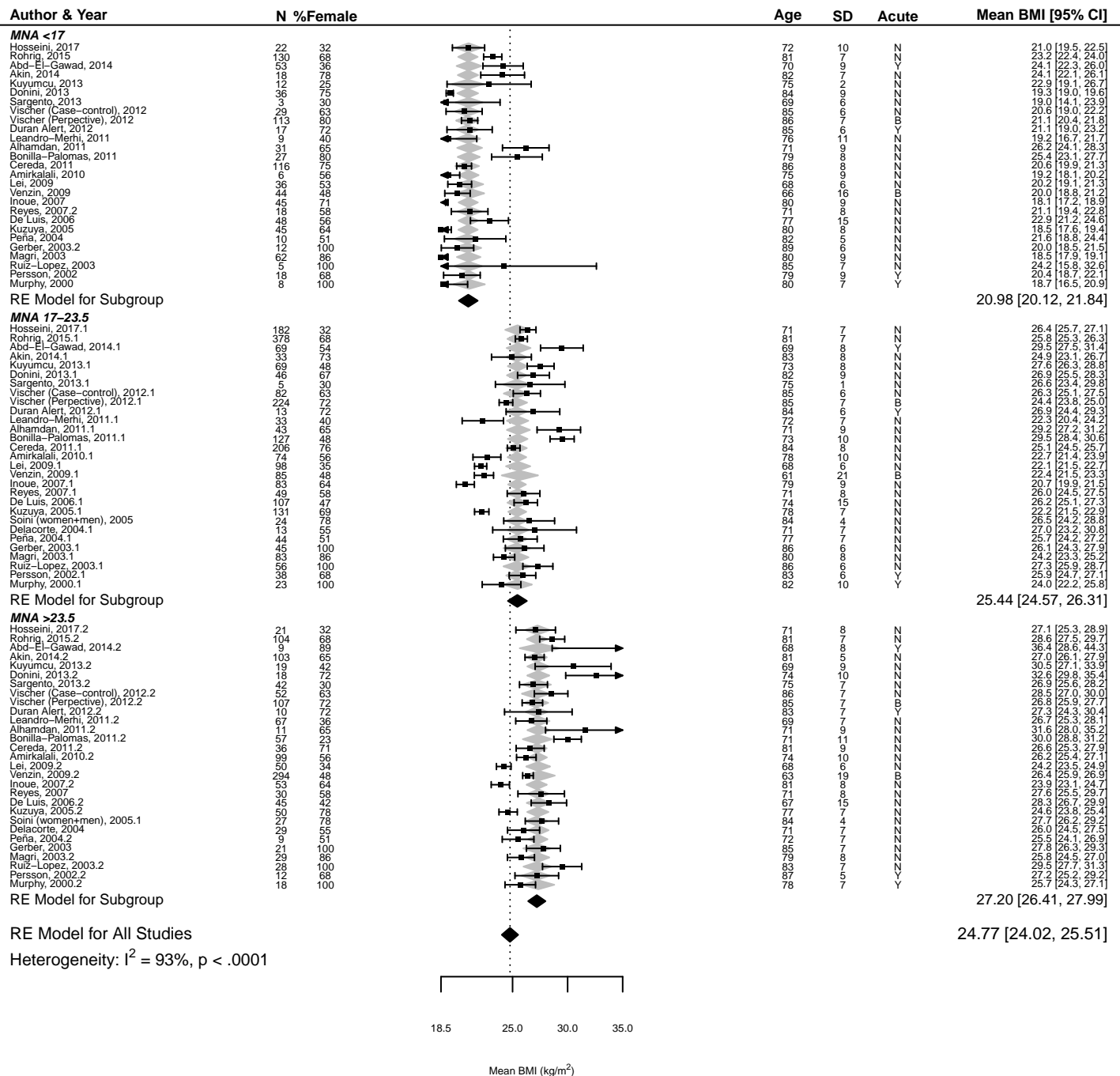

Supplement: Supplementary file 1 [file nutrients-09-00829-s001.zip › Figure S1-Forest-BMI-MNA.pdf]

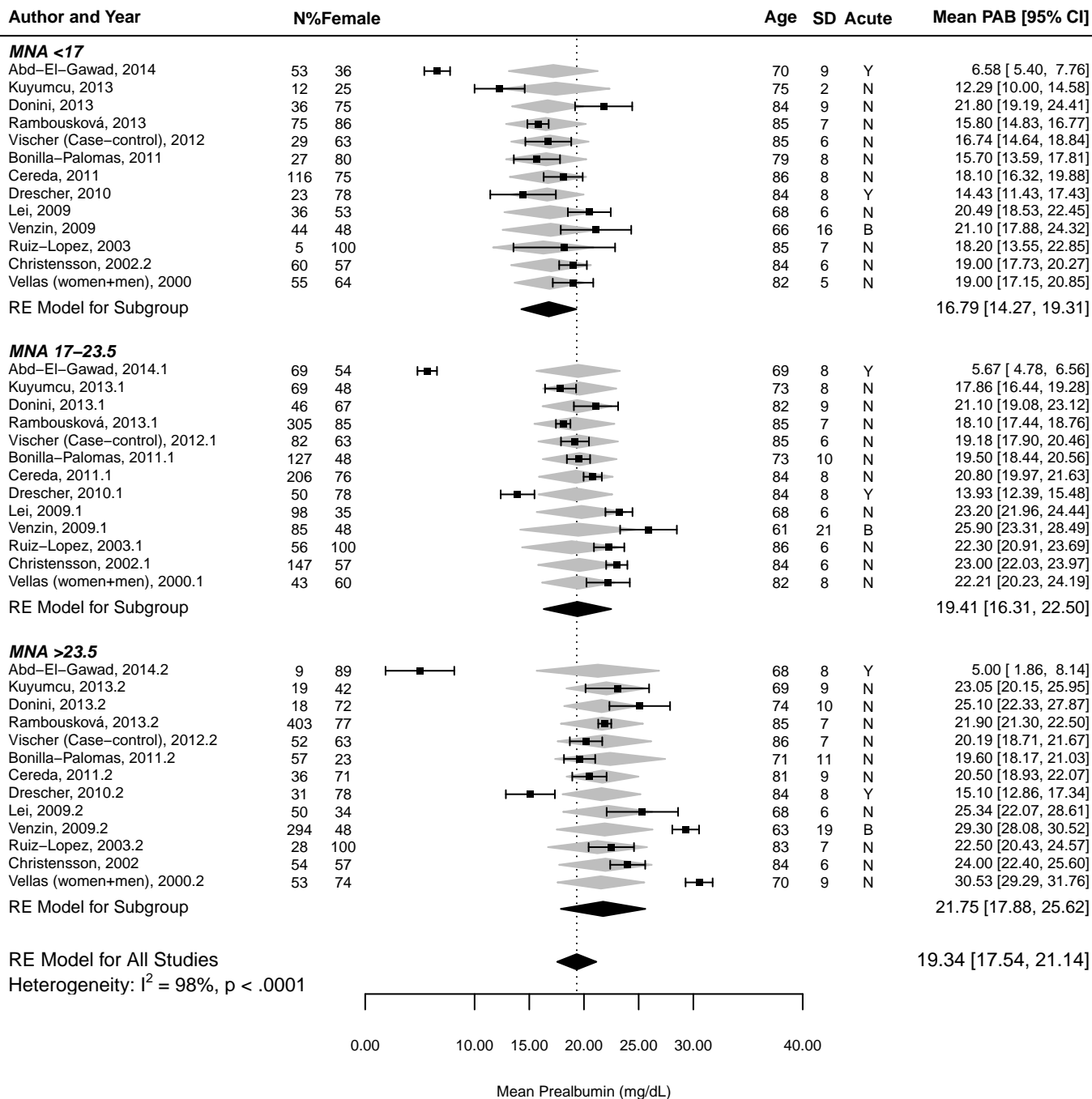

Supplement: Supplementary file 1 [file nutrients-09-00829-s001.zip › Figure S3-Forest-PAB-MNA.pdf]

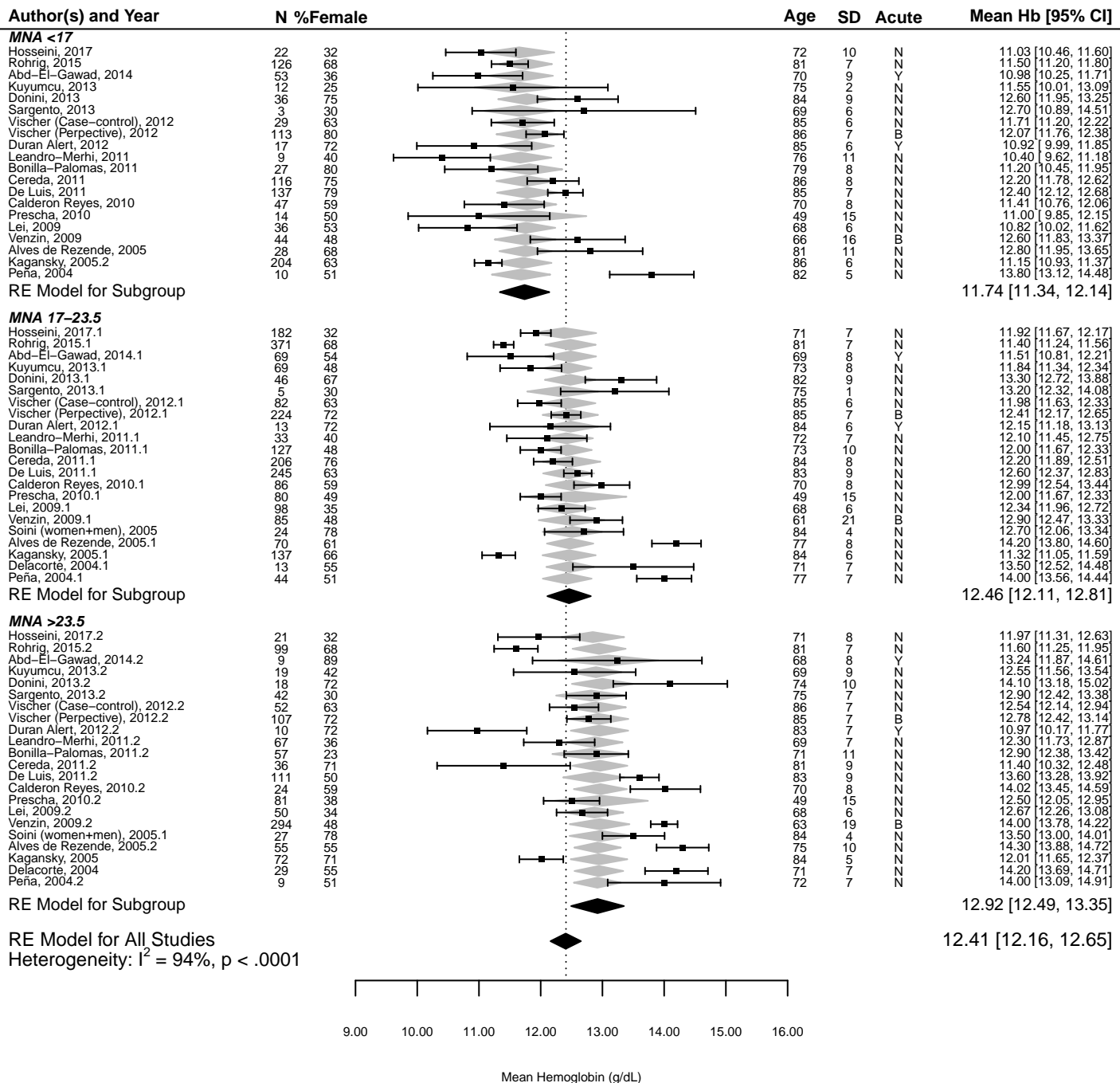

Supplement: Supplementary file 1 [file nutrients-09-00829-s001.zip › Figure S4-Forest-Hb-MNA.pdf]

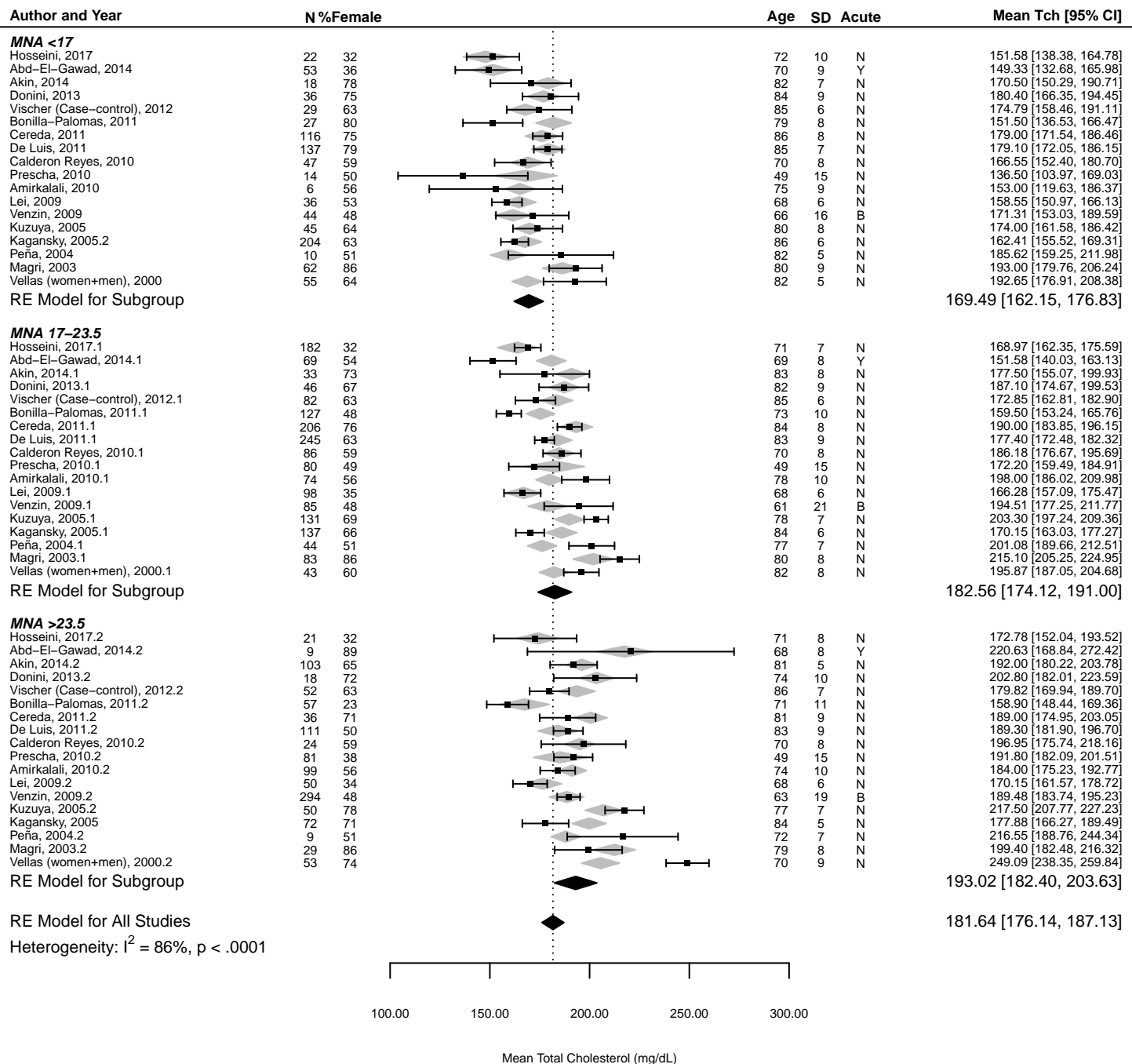

Supplement: Supplementary file 1 [file nutrients-09-00829-s001.zip › Figure S5-Forest-Tch-MNA.pdf]

Standard Error

0  
0.119  
0.238  
0.358  
0.477

Egger's test:  $t = -1.0525$ ,  $df = 91$ ,  $p = 0.2953$

-1

-0.5

0

0.5

1

Residual Value

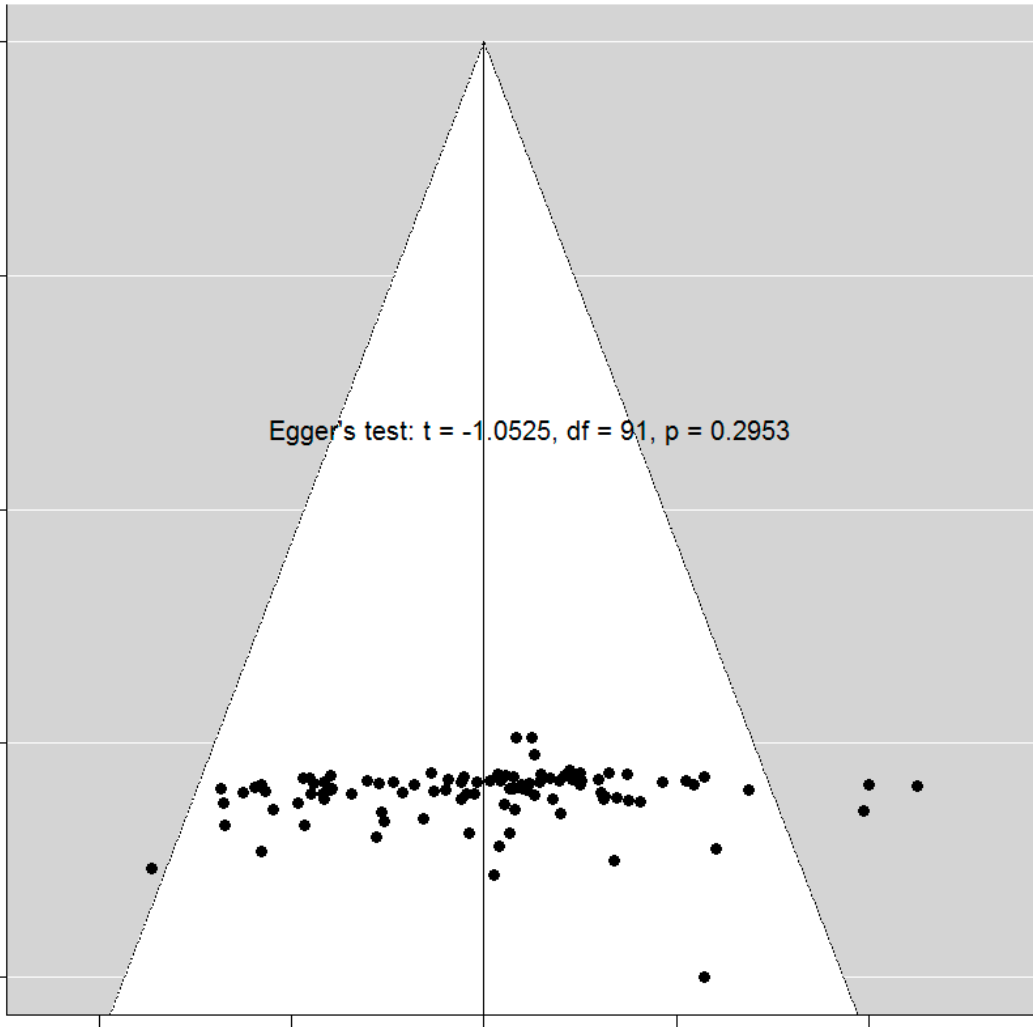

Supplement: Supplementary file 1 [file nutrients-09-00829-s001.zip › Figure S6-Funnel-alb-MNA.pdf]
